# Unlocking Plant‐Derived Potential: Regulating Microcrystalline Structure Design of High‐performance Hard Carbon Anodes via Cellulose Molecules

**DOI:** 10.1002/advs.74653

**Published:** 2026-03-09

**Authors:** Xiping Zhang, Wenhao Yang, Dan You, Ziyi Zhu, Xue Li

**Affiliations:** ^1^ National and Local Joint Engineering Research Center of Lithium‐ion Batteries and Materials Preparation Technology Key Laboratory of Advanced Battery Materials of Yunnan Province School of Metallurgical and Energy Engineering Kunming University of Science and Technology Kunming China

**Keywords:** biomass‐derived hard carbon, microcrystalline structure, sodium storage mechanism, sodium‐ion batteries

## Abstract

Biomass‐derived hard carbon (HC) stands as the leading candidate for the anode in commercial sodium‐ion batteries (SIBs). However, the effect of precursor components on the formation of HC at the molecular level breezing, and the evolution mechanism of microcrystalline carbon remains controversial. In this work, the introduction of the concept of “steric hindrance”, through reducing the steric hindrance to the growth of linear cellulose chains, which can enable the deliberate design of an ordered microcrystalline structure, resulting in significantly enhanced ion diffusion kinetics. Experimental results and molecular dynamics indicate that reduced steric hindrance can decrease the chain rigidity and internal free volume of the precursor, preventing excessive disorder, and promoting the growth of microcrystalline graphite. Additionally, highly crystalline cellulose encourages the creation of closed pores, whereas lignin and hemicellulose impede the graphitization of the carbon layer. The optimized HC anode reveals a high sodium storage capacity of 309.7 mAh/g with a high initial Coulombic efficiency (ICE) of 93.5% at 20 mA/g, excellent cycling stability over 7000 cycles at 400 mA/g. Even at ‐20°C, it still has remarkable electrochemical performance. This study offers fresh insights into the regulation of microcrystalline structures via steric hindrance engineering.

## Graphical abstract

The microcrystalline structure of hard carbon derived from green sandalwood is modulated by controlling the steric hindrance effect of the precursor, thereby significantly enhancing ion diffusion kinetics. When utilized in sodium‐ion batteries, it demonstrates excellent cycle life, exceeding 7000 cycles, as well as superior low‐temperature performance, achieving a capacity of 226.0 mAh/g at ‐20 °C.

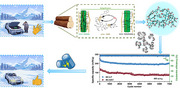

## 1 Introduction

The rapid expansion of emerging energy sources has substantially advanced the global decarbonization process. Ensuring the safety, economic feasibility, and sustainability of energy supply has become a critical challenge, thereby increasing the demand for large‐scale energy storage technologies [1]. Although lithium‐ion batteries are widely employed in energy storage applications, lithium resources face limitations such as restricted reserves, uneven geographic distribution, and price volatility [2]. In contrast, SIBs have emerged as a highly competitive complementary technology, owing to the significant advantages of abundant sodium reserves, broad geographic availability, and low raw material costs. These characteristics render SIBs particularly prospective technology for the future of energy storage systems [3, 4, 5, 6, 7, 8].

For traditional graphite anodes, the massive ionic radius of sodium ions presents significant challenges, resulting in suboptimal sodium storage performance. Therefore, it is necessary to develop other anode materials to promote the development of SIBs [9]. HC, an amorphous material distinguished by its unique disordered structure and abundant sodium storage sites, exhibits high capacity, excellent cycling stability, and favorable rate performance, positioning it as the most commercially promising anode material for SIBs [10, 11]. The selection of precursors in the synthesis of HC materials critically influences their final properties and commercial viability. When polymers and petrochemical by‐products serve as precursors, the issues such as high costs, complex processes, and substantial environmental impacts present throughout the life cycle arise [12]. In contrast, biomass‐derived HC offers notable advantages in terms of resource sustainability, environmental compatibility, cost‐effectiveness, and alignment with circular economy principles [13, 14, 15, 16]. Currently, biomass precursors have beenwidely explored, including cherry petals [17], rhododendron petals [18], magnolia grandiflora lima leaf [19], laver [20], oatmeal [21], and litchi pericarp [22].

Plants are abundant biomass resources, originating from diverse sources such as fast‐growing forests and agricultural wood waste, and are considered as to be highly renewable. Compared to other biomass‐based precursors, such as cherry petals and oatmeal, plants serve as a highly cost‐effective raw material, offering high yield and reduced carbon emissions during the fabrication process. These attributes align well with the principles of green chemistry and the demands of large‐scale industrial production. Furthermore, plants possess a distinctive natural hierarchical porous structure: at the macroscopic scale, they consist of longitudinally aligned conduits responsible for water transport and fiber cells that create micron‐sized through‐channels; at the microscopic scale, nanometer‐sized pores‐such as striated pores and interstitial spaces within cellulose microfibrils are present within the cell walls. This structural complexity can be partially preserved in the following carbonization process, resulting in a multistage pore system.

For example, Tang et al. [23]. investigated effective strategies to enhance the electrochemical activity of HC by preparing lignin‐derived HC through a straightforward pre‐oxidation method. The incorporation of pseudo‐graphitic domains and closed pores leads to significant improvements in both platform capacity and kinetic performance. Similarly, Li et al. [24]. optimized the properties of biomass precursors via a two‐step acid treatment followed by thermal treatment, which facilitated microcrystalline growth and increased the closed‐pore content, thereby enhancing sodium storage capabilities. Furthermore, Liao et al. [25]. isolated and extracted amorphous components from precursors to examine their effects on sodium storage. Their findings suggest that the rational selection of biomass precursor components, such as crystalline cellulose and lignin, can augment closed‐pore content and charge transfer properties, ultimately contributing to the development of HC anode materials for SIBs with superior electrochemical performances. Nevertheless, HC anodes encounter significant challenges under high‐rate and low‐temperature conditions, primarily due to relatively slow ion diffusion kinetics, which has been a major limitation to their further development [26, 27].

Although the above strategies have achieved notable improvements in electrochemical performance, most previous studies still tend to treat the individual components of wood merely as different carbon sources. Accordingly, the regulation strategies have primarily focused on adjusting component ratios or optimizing carbonization conditions, while systematic analyses from molecular and spatial perspectives remain largely absent. In particular, the restrictive role of different biomass components on the evolution of microcrystalline structures during carbonization has not yet been adequately elucidated.

From a compositional and structural standpoint, wood‐derived biomass does not undergo simple, independent transformations during carbonization. Instead, it experiences a complex process involving the simultaneous rearrangement and crosslinking of cellulose, hemicellulose, and lignin [28]. Among these components, lignin consists of phenylpropane units interconnected into a 3D network with a high degree of molecular branching and configurational disorder. Hemicellulose is a branched polysaccharide composed of various pentoses (e.g., xylose) and hexoses (e.g., glucose), featuring a complex and randomly distributed structure that is often covalently crosslinked with lignin. In contrast, cellulose is composed of linear long chains formed by glucose units, exhibiting a regular and symmetric structure with an intrinsic tendency to form ordered orientations through intermolecular hydrogen bonding and van der Waals interactions. These non‐linear components are widely distributed among cellulose chains and generate pronounced steric hindrance during thermal decomposition. This steric effect not only disrupts the directional alignment of cellulose chains during carbonization [29, 30], but also leads to the formation of irregular carbon fragments after carbonization, such as short‐range disordered aromatic clusters, which become embedded between cellulose‐derived lamellar structures and impede the stacking of microcrystalline layers [31]. Meanwhile, the mutual entanglement and filling of multiple components occupy the effective internal volume of the biomass, suppressing carbon framework contraction and the release of volatile species, thereby hindering pore formation [32]. As a consequence, the resulting hard carbon typically exhibits a highly disordered structure, reduced structural stability, restricted ion diffusion, a decreased number of effective active sites, and aggravated surface side reactions, ultimately leading to shortened cycling life, deteriorated rate capability, and reduced specific capacity as well as initial coulombic efficiency. Notably, this precursor‐configuration‐induced “steric hindrance effect” has not been explicitly proposed or systematically discussed in previous studies on wood‐derived hard carbon.

Based on these considerations, this work proposes a structural evolution strategy centered on steric hindrance regulation engineering. By weakening the steric constraints imposed by complex crosslinked structures on the orientation and stacking of cellulose chains, cellulose is enabled to form microcrystalline structures with higher orientation and denser packing during carbonization, while simultaneously constructing a sodium‐storage architecture dominated by closed pores [33]. This provides a structural basis for achieving high capacity, high rate capability, long cycling stability, and superior low‐temperature performance in biomass‐derived hard carbon anodes.

## 2 Results and Discussion

### 2.1 Analysis of Precursor Properties

As stated in the Introduction, the molecular composition and bonding configuration of lignocellulosic biomass play a decisive role in governing structural rearrangement and ordering during the carbonization process. In particular, highly crosslinked and branched components can induce pronounced steric effects, thereby restricting carbon‐layer reorganization and limiting the development of ordered microcrystalline domains. Therefore, prior to the analysis of carbon microstructure and electrochemical performance, it is necessary to clarify how the pretreatment selectively alters the composition and structural characteristics of the precursor.

**FIGURE 1:**
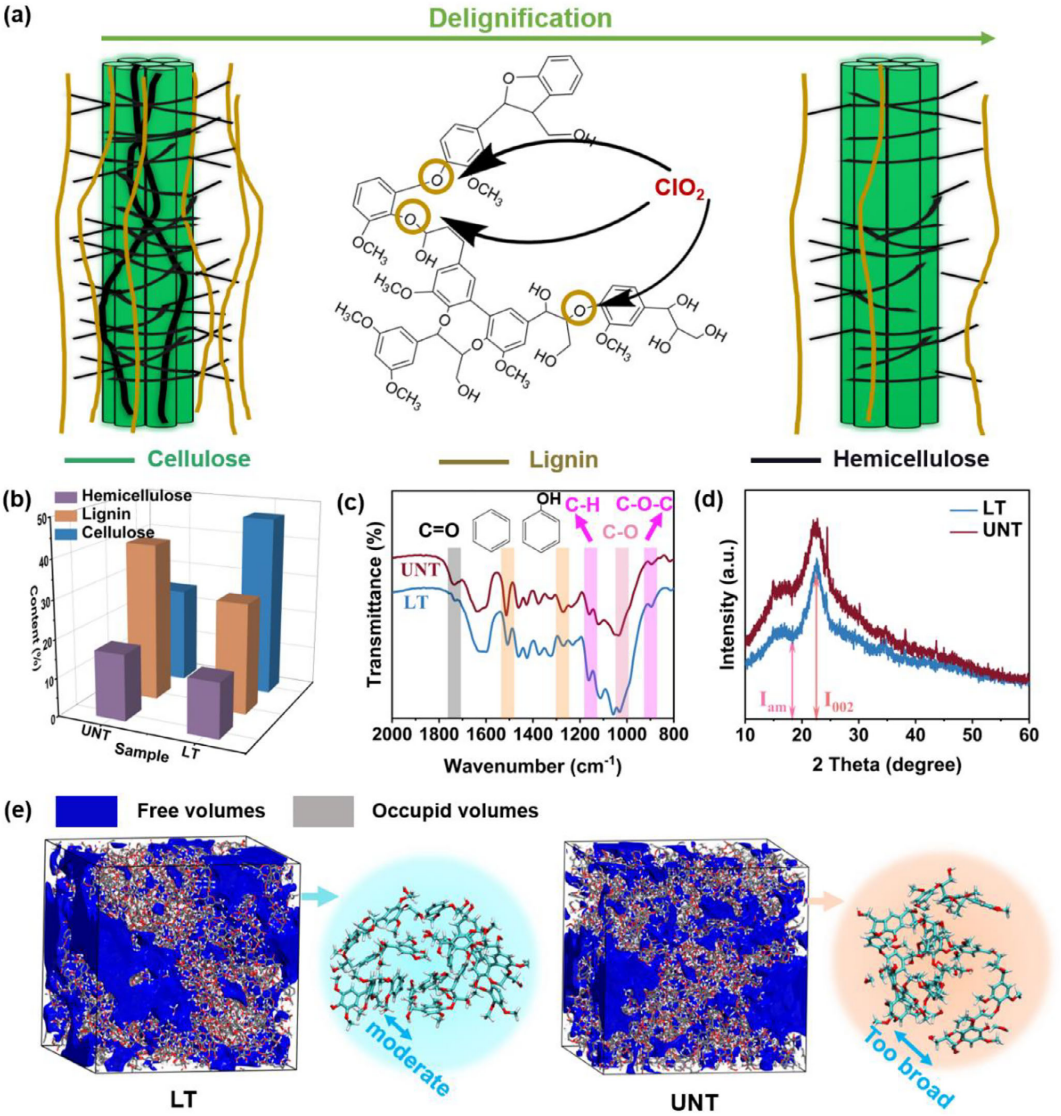
(a) Precursor modification scheme. (b) The content changes of cellulose, lignin and hemicellulose. (c) FTIR spectra. (d) XRD patterns. Calculation model of free volume for (e) LT and UNT.

As shown in Figure 1a, the ClO_2_ generated from sodium chlorite and acetic acid selectively cleaves the β─O─4 bonds in lignin molecules, enabling precise removal of lignin. Whereas during the hydrothermal reaction, the self‐ionization of water is enhanced under high‐temperature and high‐pressure conditions, leading to an increased concentration of H^+^ in the system. These H^+^ act as in situ generated acid catalysts and facilitate the occurrence of hydrolysis reactions. In addition, owing to the highly branched and amorphous structural characteristics of hemicellulose, its chemical and thermal stability is lower than that of cellulose. As a result, ester and ether linkages in hemicellulose are more susceptible to cleavage under the action of H^+^/OH^−^ species, making hemicellulose more readily hydrolyzed [34, 35]. In contrast, cellulose exhibits a more robust hydrogen‐bonding network and therefore shows stronger resistance to hydrothermal degradation; it is difficult to decompose below 200°C, allowing it to largely retain its structural integrity under these conditions [36, 37]. The modified precursor is designated as LT, whereas the untreated precursor is referred to as UNT. Changes in the contents of cellulose, lignin, and hemicellulose before and after precursor modification are quantified using the National Renewable Energy Laboratory (NREL) method, as presented in Figure 1b. Compared to UNT, LT exhibited a significant reduction in the proportions of lignin and hemicellulose and an increase in cellulose content, indicating that part of the lignin and hemicellulose was removed.

The alterations in the molecular structure are analyzed using Fourier transform infrared spectroscopy (FTIR), as illustrated in Figure 1c. In the modified LT, the intensity of the characteristic absorption peaks associated with lignin is markedly diminished. Specifically, the aromatic ring skeleton vibration at 1510 cm^−1^ and the aromatic ring methoxy C─O stretching vibration at 1270 cm^−1^ exhibit reduced intensities, indicating effective degradation or dissolution of the lignin structure. Concurrently, the absorption band at 1730 cm^−1^, which can be attributed to the carbonyl (C═O) stretching vibration in hemicellulose, diaplays a decreasing trend, confirming the efficient removal of hemicellulose during the pretreatment. Conversely, the characteristic absorption peaks of cellulose showed relative enhancement. Specifically, the intensity of the β‐glycosidic bond vibration peak at 895 cm^−1^, the antisymmetric stretching vibraion peak of the C─O─C glycosidic ring at 1160 cm^−1^, and the C─O stretching vibration peak at 1030 cm^−1^ all increases, further corroborating the relative enrichment of cellulose following modification. Changes in cellulose crystallinity between UNT and LT samples were further validated through x‐ray diffraction (XRD) analysis. As depicted in Figure 1d, the relative crystallinity index (CrI) of cellulose is according to equation (1) [38]:

(1) $$C_{r}I=\frac{I_{002}-I_{am}}{I_{002}}\;\times 100$$

I_002_ is the maximum intensity of the (002) diffraction peak in the formula, which corresponds to both crystalline and amorphous zones, and I_am_ is the minimum intensity close to 2θ = 18°, which indicates the amorphous region. According to the calculated [39], UNT's relative crystallinity is 34.9%, whereas LT's rises to 50.7%, which is consistent with the NREL result and FTIR spectral analysis. Therefore, the pretreatment effectively removed lignin and hemicellulose and enhanced the relative cellulose content. In lignocellulosic biomass, hemicellulose, cellulose, and lignin typically decompose within the temperature ranges of approximately 200–315 °C, 315–400 °C, and 350–500 °C, respectively [40]. As shown in the thermogravimetric (TG) analysis results in Figure S1, the slight difference in mass loss between LT and UNT at the low‐temperature stage (from the initial temperature to ∼220°C) can be attributed to moisture evaporation. In contrast, no pronounced difference in mass loss is observed in the high‐temperature region, because the primary devolatilization of biomass precursors is generally completed below 500°C, and the residual mass at higher temperatures mainly corresponds to the formation of thermally stable carbon [41]. Based on this analysis, the differences in the TG curves within the low and intermediate‐temperature decomposition regions can qualitatively reflect changes in precursor component composition after pretreatment, thereby providing qualitative evidence for the effective removal of hemicellulose and lignin.Additionally, LT exhibits a higher thermal weight loss rate in the high‐temperature region. This observation indicates that following the partial elimination of lignin and hemicellulose, the pyrolytic reactivity increases, the required activation energy decreases, and pyrolysis becomes more readily achievable. Analysis suggests that lignin and hemicellulose, as amorphous polymers with 3D crosslinked structures, possess complex spatial configurations that introduce significant steric hindrance during the pyrolysis process. This hindrance restricts the mobility, rearrangement, and aromatization of the carbon skeleton. Consequently, partial removal of lignin and hemicellulose can effectively reduce these inhibitory effects, resulting in favorable conditions for the structural reorganization of the carbon skeleton and the orderly arrangement. To confirm the reduction of steric hindrance during this process, differential scanning calorimetry (DSC) tests were performed. As illustrated in Figure S2, the glass transition temperature (T_g_) of the LT precursor decreased to 310.885°C compared to 315.848°C for the UNT precursor [42], indicating a reduction in molecular chain rigidity and thereby confirming the successful alleviation of steric hindrance [43]. This conclusion is further supported by FTIR spectroscopy of the LT and UNT precursors (Figure S3). The FTIR spectra reveal an intensified hydroxyl stretching vibration peak that shifts to lower wavenumbers (from 3441.35 to 3423.99 cm^−1^), which suggests an increase in hydrogen bond content within the sample [44]. These observations indirectly corroborate the effective reduction of steric hindrance [45, 46]. Furthermore, the effect of various precursor components on free volume was investigated by molecular dynamics simulations. As shown in Figure 1e and Table S1, free volume visualization obtained via GROMACS simulations indicates that the free volume of LT is smaller than that of UNT. This reduction is primarily attributed to the removal of lignin/hemicellulose, which causes a more compact molecular structure that promotes the formation of graphite microcrystals and improves overall structural order.

### 2.2 Structure Analysis of HC

**FIGURE 2:**
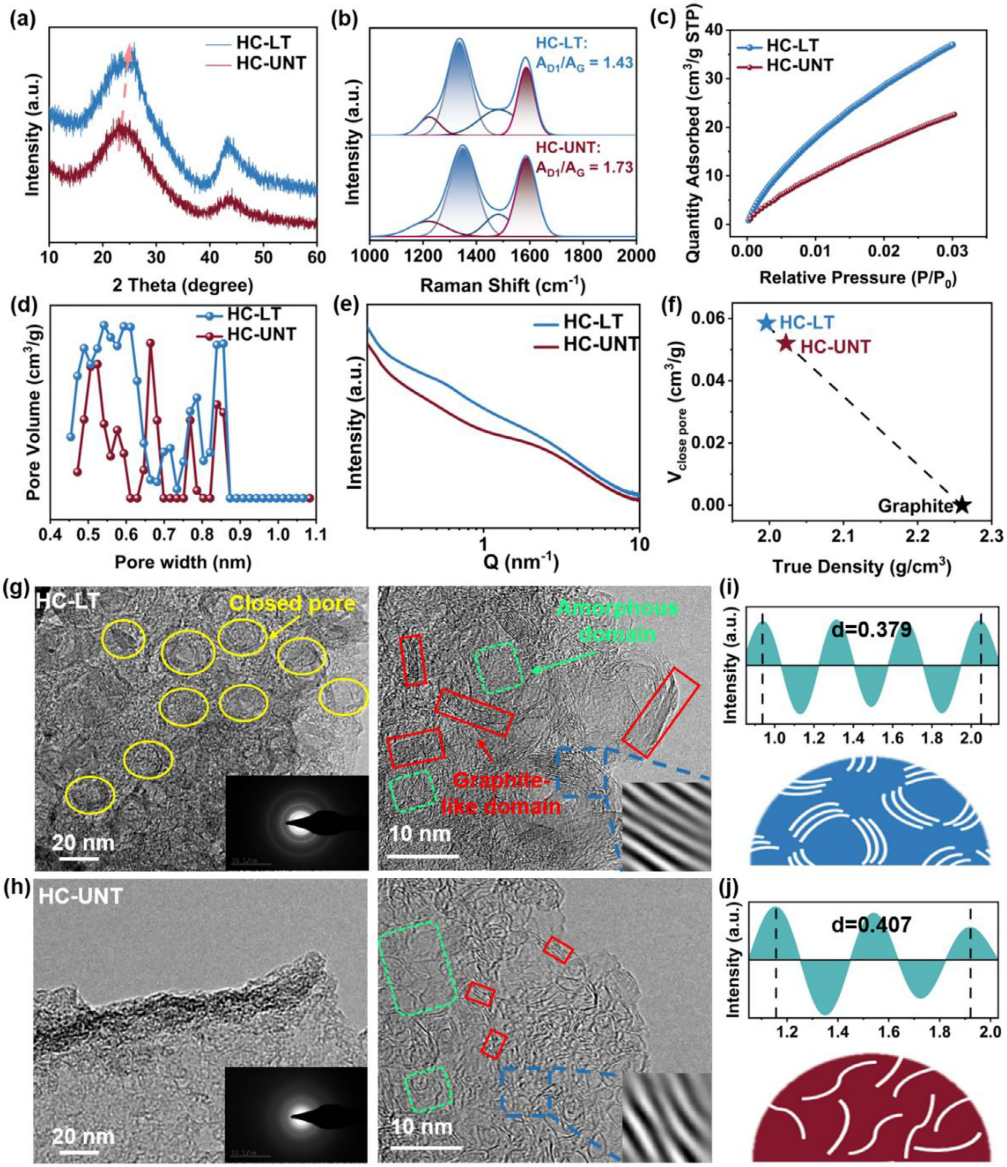
(a) XRD patterns, (b) Raman spectra, (c) CO_2_ adsorption and desorption curves, (d) the corresponding pore size distribution, (e) SAXS curves, and (f) Closed pore volume of HC‐LT and HC‐UNT. HRTEM images of (g) HC‐LT and (h) HC‐UNT. Carbon layer spacing and schematic diagram of microstructure for (i) HC‐LT and (j) HC‐UNT.

To explore the evolution of microstructure XRD and Raman spectroscopy were conducted on HC‐UNT and HC‐LT. As shown in Figure 2a, both samples exhibit two broad amorphous diffraction peaks centered at approximately 24° and 43°, which can be assigned to the (002) and (100) planes, respectively, representing the typical structural features of hard carbon. Compared with HC‐UNT, the (002) peak of HC‐LT shifts toward a higher diffraction angle, indicating a reduced interlayer spacing, which is associated with the growth of graphitic‐like microcrystalline domains and local structural ordering. The interlayer interactions within these microcrystallites are primarily governed by van der Waals forces. With the growth of microcrystallites, the lateral size of the graphene layers increases (Table S2 shows that the average carbon‐plane width L_a_ increases from 2.2 nm for HC‐UNT to 2.6 nm for HC‐LT), leading to a reduction in structural defects. Meanwhile, the parallel alignment between adjacent layers is improved, resulting in an increased effective overlap area and a substantial enhancement of the cumulative van der Waals interactions. Thermodynamically, this favors the stacking of carbon layers and drives the interlayer spacing to contract toward a more stable structural configuration. Raman spectroscopy is employed to characterize defect features. The spectra display five characteristic peaks: the D1 band (1350 cm^−1^, associated with disordered graphite lattice), D2 band (1620 cm^−1^, related to turbulent graphite layers), D3 band (1500 cm^−1^, indicative of short‐range sp^3^ carbon), D4 band (1200 cm^−1^, corresponding to sp^2^‐sp^3^ mixed structures), and the G band (1580 cm^−1^, representing an ideal graphite lattice) [47, 48]. Among these, the degree of disorder in the carbon structure is shown by the area ratio of the D1 to G bands (A_D1_/A_G_). As shown in Figure 2b, the microcrystalline structural optimization reduces the A_D1_/A_G_ ratio from 1.73 in HC‐UNT to 1.43 in HC‐LT, signifying an enhanced degree of graphitization.

The microporous structural characteristics of the hard carbon were evaluated by CO_2_ adsorption‐desorption measurements, as shown in Figure 2c,d. The specific surface area and micropore volume of HC‐LT are 163.76 m^2^/g and 0.04 cm^3^/g, respectively, both of which are significantly higher than those of HC‐UNT (100.36 m^2^/g and 0.02 cm^3^/g). Owing to the stronger diffusion capability of CO_2_ molecules, which enables effective probing of ultramicropores that are inaccessible to conventional N_2_ at 77 K, these results indicate a pronounced increase in the ultramicropore population of HC‐LT. Meanwhile, N_2_ adsorption‐desorption measurements (Figure S4a,b) show that HC‐LT also exhibits higher specific surface area (89.3 m^2^/g) and pore volume (0.073 cm^3^/g) than HC‐UNT (44.6 m^2^/g and 0.049 cm^3^/g), suggesting the concurrent development of pore structures in the larger micropore and mesopore size ranges. Although CO_2_ and N_2_ adsorption respond to different pore size regimes, the consistent trends in specific surface area and pore volume revealed by both techniques indicate that the pore structure optimization of this material is not confined to a single scale but rather involves a synergistic evolution across multiple length scales, spanning from ultramicropores to mesopores.Theoretically, the steric hindrance imposed by lignin and hemicellulose inhibits pore formation by occupying potential pore space, impeding the escape of volatile compounds and restricting the shrinkage of the carbon skeleton. The removal of these components alleviates such constraints, thereby enabling the HC to develop a greater number of structurally diverse pores during pyrolysis. This hierarchical porous architecture not only increases the interfacial contact area between the electrode and electrolyte, providing more sites for sodium‐ion adsorption, but also facilitates more efficient ion transport by offering smoother diffusion pathways and increased pore volume for sodium‐ion storage [49, 50].

Previous research has demonstrated that the closed pore structure is essential for sodium‐ion storage. Consequently, small‐angle x‐ray scattering (SAXS) analysis was employed to further investigate the characteristics of the closed‐pore structure. As illustrated in Figure 2e, a distinct scattering peak appears at Q ≈ 1 nm^−1^, which is indicative of the presence of closed pores [51]. The scattering intensity observed for HC‐LT exceeds that of HC‐UNT, indicating a higher percentage of closed pores [26]. Furthermore, the brightness and intensity of the 2D SAXS image (Figure S5) reveal that the HC‐LT sample contains a substantial number of isotropic pores, implying a diverse porosity that favors the formation of closed pores [24, 48]. Additionally, true density is a critical parameter for assessing the internal closed pore structure of carbon materials. Due to its small kinetic diameter and van der Waals radius, helium can penetrate most open pores but is unable to access closed pores, making it a useful probe for determining the true density of materials and thereby providing supplementary validation of SAXS findings [52]. To ensure the reliability of true density analysis, ideal graphite with a true density of 2.26 g/cm^3^ serves as a benchmark for other materials, which has perfect crystalline layered materials devoid of closed pores [53, 54, 55]. The closed pore volume of the different HCs can be calculated using equation (2):

(2) $$V_{Closed\;Pore}=\frac{1}{\rho_{ture}}\;-\frac{1}{2.26}$$

As illustrated in Figure 2f, the true density of HC‐LT (1.996 g/cm^3^) is lower than HC‐UNT (2.0223 g/cm^3^), indicating a higher closed pore volume content (0.0585 cm^3^/g).

Figure S6 presents scanning electron microscope (SEM) images of HC‐UNT and HC‐LT, revealing minimal differences in macroscopic morphology, particle sizes between 5 and 20 µm. Microstructural variations were further examined using high‐resolution transmission electron microscopy (HRTEM), as depicted in Figure 2g,h, and Figure S7. HC‐LT consists of both amorphous and graphitic‐like regions, whereas the graphitic‐like microcrystals in HC‐UNT are shorter and exhibit greater disorder. Additionally, the interlayer spacing of the carbon layers significantly decreased from 0.407 nm in HC‐UNT to 0.379 nm in HC‐LT (Figure 2i,j). An increased content of crystalline cellulose appears to facilitate carbon layer rearrangement, thereby promoting the growth and curling of graphitic‐like microcrystalline regions and leading to the formation of more closed pore.

### 2.3 Electrochemical Performance

**FIGURE 3:**
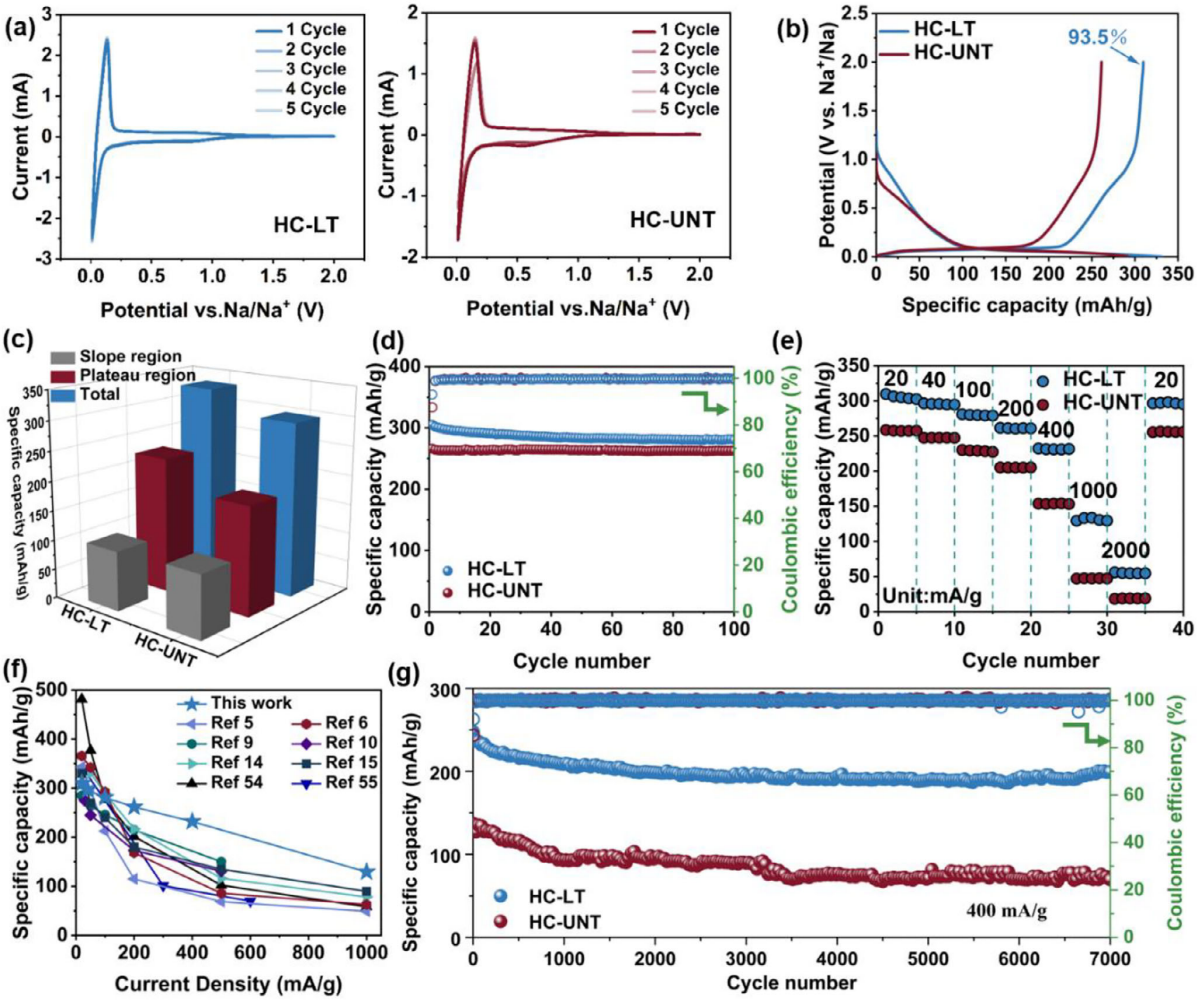
CV curves of the (a) HC‐LT electrode and HC‐UNT electrode. (b) GCD curves (20 mA/g), (c) Capacity proportion of different potential ranges, (d) Cycle performance (20 mA/g), (e) Rate performance of the HC‐LT electrode and HC‐UNT electrode. (f) Performance comparison with some biomass‐derived HC. (g) Cycle performance (400 mA/g).

Figure 3a presents the cyclic voltammetry (CV) curves of HC‐LT and HC‐UNT recorded at a scan rate of 0.1 mV s^−1^. In the initial cycle, the characteristic current peak is observed at approximately 0.55 V corresponds to the decomposition of the electrolyte, resulting in the formation of a solid electrolyte interphase (SEI). This peak disappears in the subsequent cycles, indicating that SEI formation predominantly occurs during the initial reaction stage. Additionally, the prominent redox peak near 0.01 V is attributed to the reversible intercalation and deintercalation of sodium ions within the graphitic‐like microcrystalline layers. Compared with HC‐UNT, HC‐LT exhibits a higher redox current peak, suggesting enhanced electrochemical activity and reduced charge transfer resistance.

In the constant current charge‐discharge tests, the HC‐UNT electrode possess a reversible capacity of only 261.2 mAh/g (Figure 3b), whereas the HC‐LT electrode achieves a higher capacity of 309.7 mAh/g, along with a high ICE of 93.5%. As illustrated in Figure 3c, the capacity difference between the two electrodes primarily arises from the contribution of the low potential plateau region, in which HC‐LT can deliver higher capacity of 230.1 mAh/g than that of HC‐UNT (184.2 mAh/g). Besides, the HC‐LT electrode shows superior specific capacity and excellent cycling stability in the current density range of 20 to 1000 mA/g. Specifically, as shown in Figure 3d, the HC‐LT electrode maintains a capacity of 280.4 mAh/g after 100 cycles at 20 mA/g. Even when the current density is increased to 1000 mA/g, the HC‐LT electrode sustains a reversible capacity of 133.2 mAh/g (Figure 3e), whereas the HC‐UNT electrode exhibits only 47.0 mAh/g. When the current density is returned to 20 mA/g, the capacity HC‐LT can recover to 296.6 mAh/g, corresponding to 95.8% of the initial capacity, indicating excellent stability and high reversibility during charge‐discharge cycling. Compared to other HC, the HC‐LT electrode also displays a competitive advantage (Figure 3f; table S3). Furthermore, the HC‐LT anode presents impressive cycling stability at high rates (Figure 3 g; Figure S8), retaining a high capacity ratio of 89.3% after 1200 cycles at 1000 mA/g and 80.5% after 7000 cycles at 400 mA/g (the average capacity decay rate is approximately 0.0028% per cycle), significantly outperforming the HC‐UNT electrode (51.3% capacity retention with an average fading rate of ∼0.007% per cycle). To assess potential commercial applicability, a full cell was assembled using a Na_3_V_2_(PO_4_)_3_ (NVP) cathode and the HC‐LT anode with an N/P ratio of 1.15 (Figure S9a), successfully powering a “KUST” lamp (Figure S9b). As depicted in Figure S9c, the full cell exhibits a capacity retention of 90.2% after 100 cycles within a voltage window of 2.0–3.8 V, along with favorable rate performance (Figure S9d).

The greatly enhanced overall performances are attributed to the optimization of the microcrystalline structure resulting from the reduction of steric hindrance. Specifically, an optimal carbon layer spacing facilitates the accommodation of a greater number of sodium ions. Additionally, the bent graphite microcrystalline structure promotes the formation of more topologically closed pore structures, which provides ample space for the reversible incorporation of metallic sodium clusters.

### 2.4 Kinetics Performance

**FIGURE 4:**
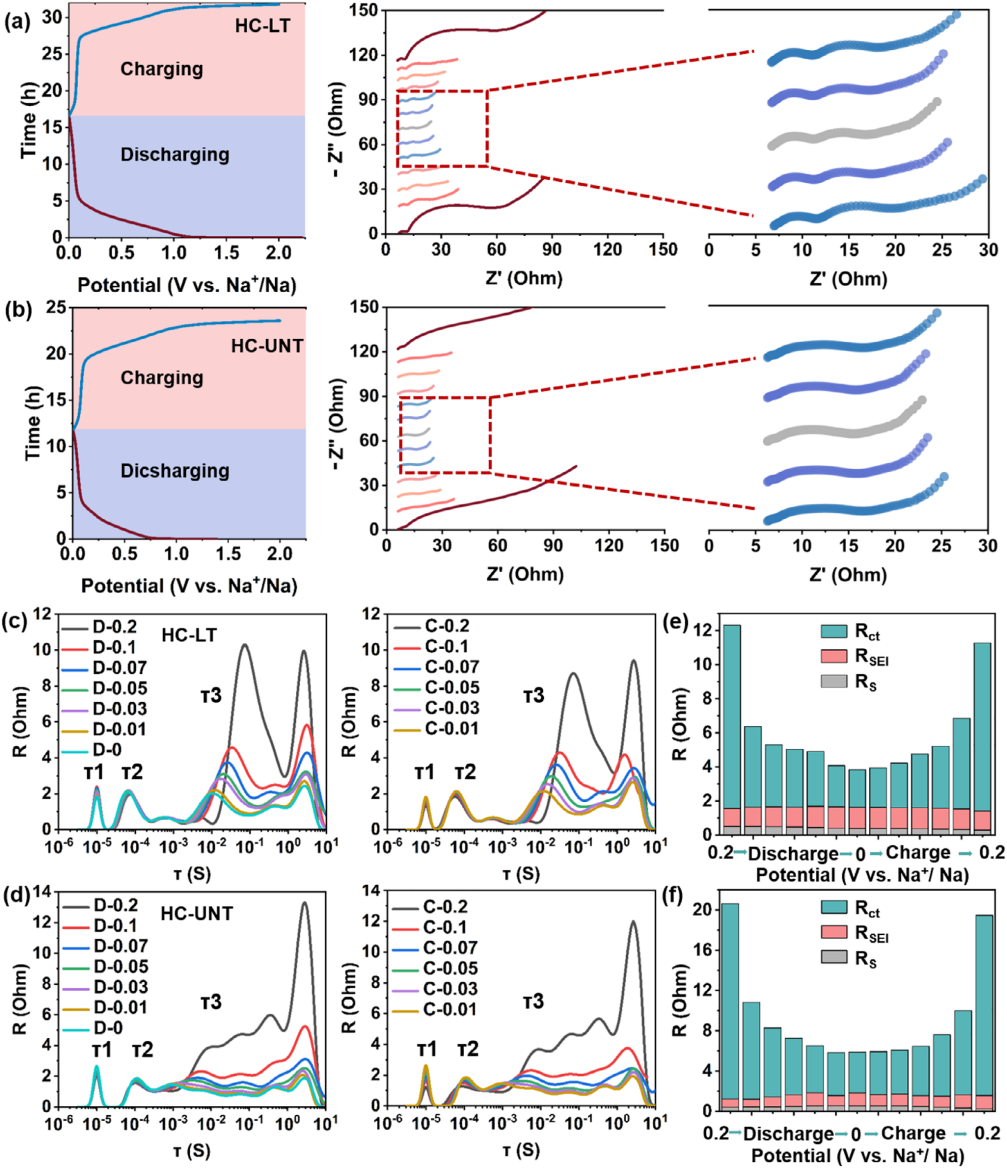
Potential‐time curves and the corresponding In situ impedance curves of (a) HC‐LT electrode and (b) HC‐UNT electrode. DRT spectra of (c) HC‐LT electrode and (d) HC‐UNT electrode. The corresponding resistance fitting results of (e) HC‐LT electrode and (f) HC‐UNT electrode.

As illustrated in Figure 4a,b, in situ electrochemical impedance spectroscopy (EIS) measurements were conducted on HC‐LT and HC‐UNT. The Nyquist plot comprises three distinct regions: the solution resistance (R_S_) observed in the high‐frequency region, which reflects the resistance associated with ion conduction; the impedance of the solid electrolyte interphase SEI film (R_SEI_), representing the conduction resistance of the SEI layer to sodium ions along with its corresponding capacitive behavior; the charge transfer impedance (R_ct_) located in the middle to high‐frequency region, corresponding to the resistance of the charge transfer reaction at the HC/electrolyte interface and its non‐ideal capacitive characteristics; and finally, the Warburg impedance in the low‐frequency region, which is related to the diffusion process of ions within the bulk phase.

To elucidate the impedance variation trends during charging and discharging, relaxation time distribution (DRT) analysis was employed to deconvolute the EIS data. As illustrated in Figure 4c,d, the DRT spectra under both discharge and charge conditions exhibit three characteristic peaks within the time constant (τ) range of 10^−6^ to 101 s, corresponding to R_S_ (τ1,10^−6^‐10^−5^ s), R_SEI_ (τ2,10^−4^ s), R_ct_ (τ3,10^−4^‐10^−1^ s) [56, 57]. The R_S_, R_SEI_ and R_ct_ values for HC‐LT and HC‐UNT electrodes, obtained through curve fitting, are summarized in Figure 4e,f. The results indicate that R_S_ values of the two electrodes are comparable, suggesting similar electrolyte ion conduction properties. The R_SEI_ increases slightly during the charge‐discharge cycle, primarily due to the continuous formation and evolution of the solid electrolyte interphase SEI film [58]; and the R_ct_ decreases with increasing depth of discharge, reflecting enhanced electrode material conductivity and optimized electrochemical reaction kinetics resulting from sodium‐ion insertion. Notably, the R_ct_ of the HC‐LT electrode is significantly lower than that of HC‐UNT, implying that the development of the modified graphite microcrystalline structure more effectively enhances conductivity. This improvement facilitates accelerated charge transfer reactions at the electrode/electrolyte interface and reduces the electron reduction resistance of sodium ions at the HC‐LT interface, ultimately leading to higher interfacial reaction rates and superior reaction kinetics.

**FIGURE 5:**
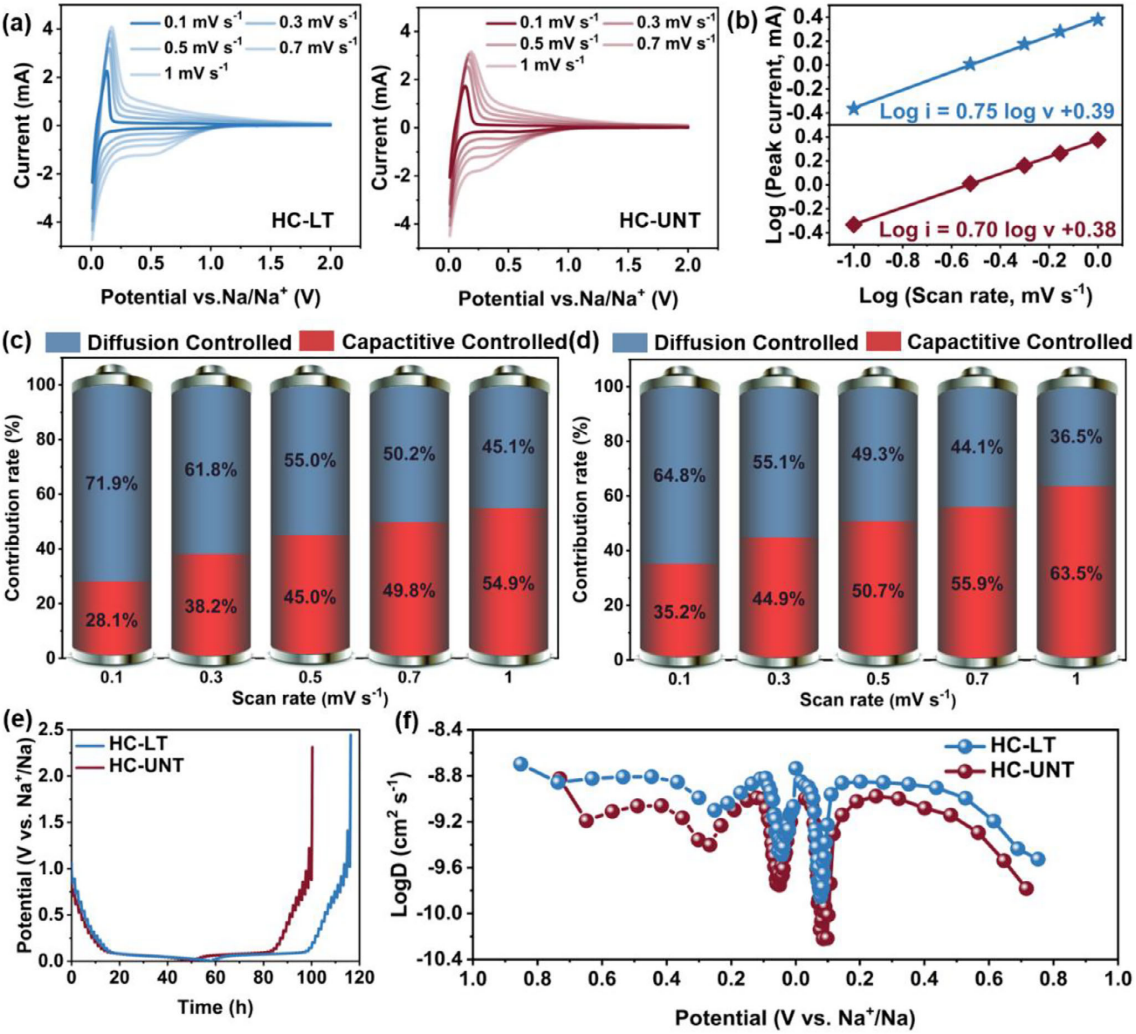
(a) CV at different scan rates, and (b) the relationship between log (i) and log (v) of the HC‐LT and HC‐UNT electrodes. Normalized percentages of capacitive‐controlled capacities at various scan rates of the (c) HC‐LT electrode and (d) HC‐UNT electrode. (e) GITT potential profiles, and (f) the apparent ion diffusion coefficients.

CV tests were conducted to investigate the kinetic characteristics. As illustrated in Figure 5a, the relationship between the peak current (i) and the scan rate (v) is described by equation (3):

(3) $$i=av^{b}$$

In the formula, a and b represent constants. When the value of b approaches 0.5, the reaction is primarily governed by diffusion control. Conversely, when b approaches 1, the behavior is dominated by capacitance. As illustrated in Figure 5b, a strong linear relationship is observed between log(v) and log(i), with the b values for HC‐UNT and HC‐LT determined from the slope to be 0.70 and 0.75, respectively. These values suggest the coexistence of sodium storage processes involving both pseudocapacitance and diffusion control mechanisms. To further quantify the contributions of these two mechanisms, Equation (4) was employed to decompose the total current:

(4) $$i=k_{1}\upsilon +k_{2}\upsilon^{1/2}$$

The current (i) can be described as the sum of a capacitive process, represented by k_1_v, and a diffusion‐controlled process, represented by k_2_v^1/2^, where k_1_ and k_2_ are constants. By plotting the linear relationship between i/v^1/2^ and v^1/2^, the values of k_1_ and k_2_ can be determined, thereby quantifying the relative contributions of the diffusion‐controlled and capacitive processes. As illustrated in Figure 5c,d, the electrochemical behavior progressively shifts toward capacitive control dominance with increasing scan rate. Quantitative analysis reveals that the capacitive contribution of the HC‐LT electrode increases from 28% at 0.1 mV s^−1%^ to 55% at 1 mV s^−1^, indicating a gradual transition in the sodium storage mechanism from insertion and pores filling to surface adsorption. Notably, the contribution ratio of the diffusion‐controlled process in the HC‐LT electrode remains consistently higher than that of HC‐UNT, which aligns with its superior platform capacity performance.

Furthermore, the diffusion coefficient ($D_{Na^{+}}$) of sodium ions in HC‐UNT and HC‐LT was investigated using the constant current intermittent titration technique to further elucidate the sodium storage behavior. As illustrated in Figure 5e, the similar profiles of the GITT curves suggest that both materials exhibit comparable sodium storage mechanisms. The diffusion coefficient $D_{Na^{+}}$ is determined based on Fick's second law [59]:

(5) $$D_{Na^{+}}=\frac{4}{\pi \tau }\;{\left(\frac{m_{b}V_{m}}{M_{B}S}\right)}^{2}{\left(\frac{\Delta E_{S}}{\Delta E_{t}}\right)}^{2}$$

Among these variables, τ (s), m_b_ (g), V_m_ (cm^3^/mol), M_B_ (g/mol), and S (m^2^/g) denote relaxation time, active mass, molar volume, molecular weight, and electrode area, respectively. ΔE_s_ and ΔE_t_ represent the potential changes occurring during the current pulse and the constant current discharge‐charge process, respectively, after the elimination of the IR drop.

Figure 5f illustrates the variation trend of the sodium‐ion diffusion coefficient ($D_{Na^{+}}$) as a function of potential. In the high potential region (>0.1 V), sodium ions are primarily stored through rapid adsorption and desorption on the HC surface or at defect sites, resulting in a higher $D_{Na^{+}}$ due to the short diffusion pathways and low resistance. As the potential decreases to 0.1 V, $D_{Na^{+}}$ declines sharply, which is attributed to the intercalation and deintercalation processes of sodium ions within the graphitic‐like microcrystalline layers. Changes in interlayer spacing and lattice stress during this process increase diffusion resistance. During closed pore filling, sodium ions diffuse into closed pores via open pores or defects, forming metallic sodium clusters. Owing to the high conductivity of metallic sodium, $D_{Na^{+}}$ exhibits a significant increase. Notably, the $D_{Na^{+}}$ of the HC‐LT electrode is higher than that of the HC‐UNT electrode, primarily due to microcrystalline structural regulation induced by enhanced steric hindrance, which optimizes the transport pathways and kinetic environment for sodium‐ion diffusion. The graphitic‐like domain structure not only provides a greater number of insertion sites but also establishes smoother transmission channels. These findings are consistent with the observed improvements in platform capacity, total capacity, and rate performance of the HC‐LT electrode, all of which depend on rapid and efficient sodium‐ion transport kinetics.

### 2.5 Sodium Storage Mechanism

**FIGURE 6:**
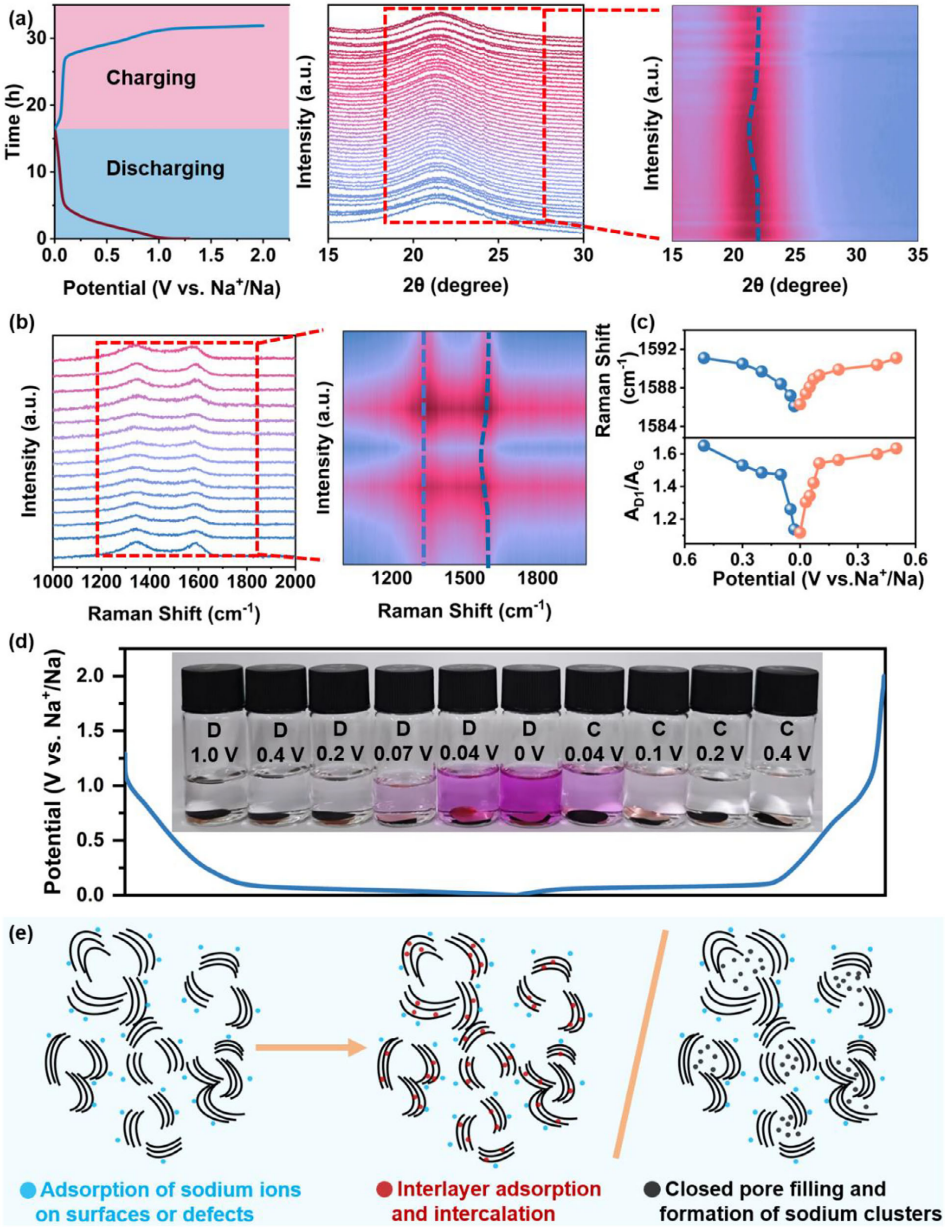
(a) In situ XRD patterns. (b) In situ Raman spectra, and (c) The corresponding G band position and the change trend of A_D1_/A_G_ with potential. (d) Color reaction. (e) Schematic diagram of sodium storage mechanisms.

To systematically investigate the structural changes and electrochemical reaction mechanisms of the HC‐LT electrode during cycling processes, in situ XRD and in situ Raman spectroscopy were employed. As illustrated in Figure 6a, no significant shift is observed in the position of the (002) diffraction peak in the XRD pattern during the initial discharge stage, indicating that the interlayer spacing of the carbon can remain stable. This observation aligns with the characteristics of sodium‐ion adsorption storage behavior on the electrode surface. Upon entering the low potential region (<0.1 V) during discharge, the (002) diffraction peak shifts toward lower angles, indicating that sodium‐ion insertion induces an expansion of the carbon interlayer space [60, 61, 62]. Concurrently, the diffraction peak broadens and the intensity diminishes, which may be attributed to the combined effects of closed pore filling and the formation of sodium clusters. The in situ Raman spectra of HC‐LT, presented in Figure 6b, reveal that the D1 band gradually broadens and the intensity decreases as discharge progresses, while the peak position remains unchanged. The phenomenon is attributed to sodium‐ion adsorption at active sites, inhibiting the breathing vibrations of carbon rings at defect sites and edges.With further potential reduction, sodium‐ion insertion into the graphite layers increases electron density, weakens π–π* interactions, and induces a continuous redshift of the G band (Figure 6c). Additionally, sodium‐ion aggregation within closed pores leads to local structural rearrangements, resulting in a marked decrease in the intensity ratio of the D1 to G bands (A_D1_/A_G_) in the low potential region. Upon completion of charging, both the (002) diffraction peak in the XRD pattern and the D1/G bands in the Raman spectrum almost return to their initial states, demonstrating the excellent reversibility of sodium‐ion storage behavior.

Due to the unique characteristics of the closed pore structure‐, namely its sealing and nanoscale dimensions, it is challenging to directly verify the sodium‐ion filling behavior using conventional electrochemical tests and in situ characterization techniques. To address this limit, the specific reaction between sodium and ethanol solvent was employed to further confirm the presence of quasi‐metallic sodium clusters via the color change of an indicator. As illustrated in Figure 6d, electrodes at different charge states were immersed in an ethanol solution containing 1% phenolphthalein. In the high potential region, the color of the ethanol‐phenolphthalein solution exhibits no significant change; however, as the potential decreased, the solution gradually turns deep red. This color change results from the reaction of sodium with ethanol to form alkaline sodium ethoxide, which induces discoloration of the indicator, thereby confirming the existence of quasi‐metallic sodium clusters in the low‐potential platform region (HC‐LT) and supporting the sodium storage mechanism based on closed pore filling. The ionic conductivity measurements of the solution post‐reaction are presented in Figure S10a. The ionic conductivity shows minimal variation in the high potential region but increased progressively during the discharge process, consistent with the observed color development. Under identical conditions, the comparisons of the color changes in ethanol of fresh electrodes, electrodes discharges to 0 V, and metallic sodium in ethanol‐phenolphthalein solution (Figure S10b) further corroborates these findings. Additionally, SAXS analysis demonstrated that sodium ions occupy the pores and intercalate within the interlayers of graphitic‐like microcrystals, leading to a marked decrease in the intensity of pore‐related scattering signals (Figure S11). HRTEM images reveal characteristic particles corresponding to quasi‐metallic sodium clusters (Figure S12), thereby validating the pore‐filling mechanism from a structural perspective. In summary, the sodium storage mechanism of the HC‐LT anode adheres to an “adsorption‐intercalation/pore filling” model (Figure 6e): the capacity observed in the high potential slope region is attributed to sodium‐ion adsorption on surface or defect sites, whereas the capacity in the low potential plateau region arises from the synergistic effects of graphitic‐like structure intercalation, closed pore filling, and quasi‐metallic sodium cluster formation.

### 2.6 Low‐Temperature Performance

**FIGURE 7:**
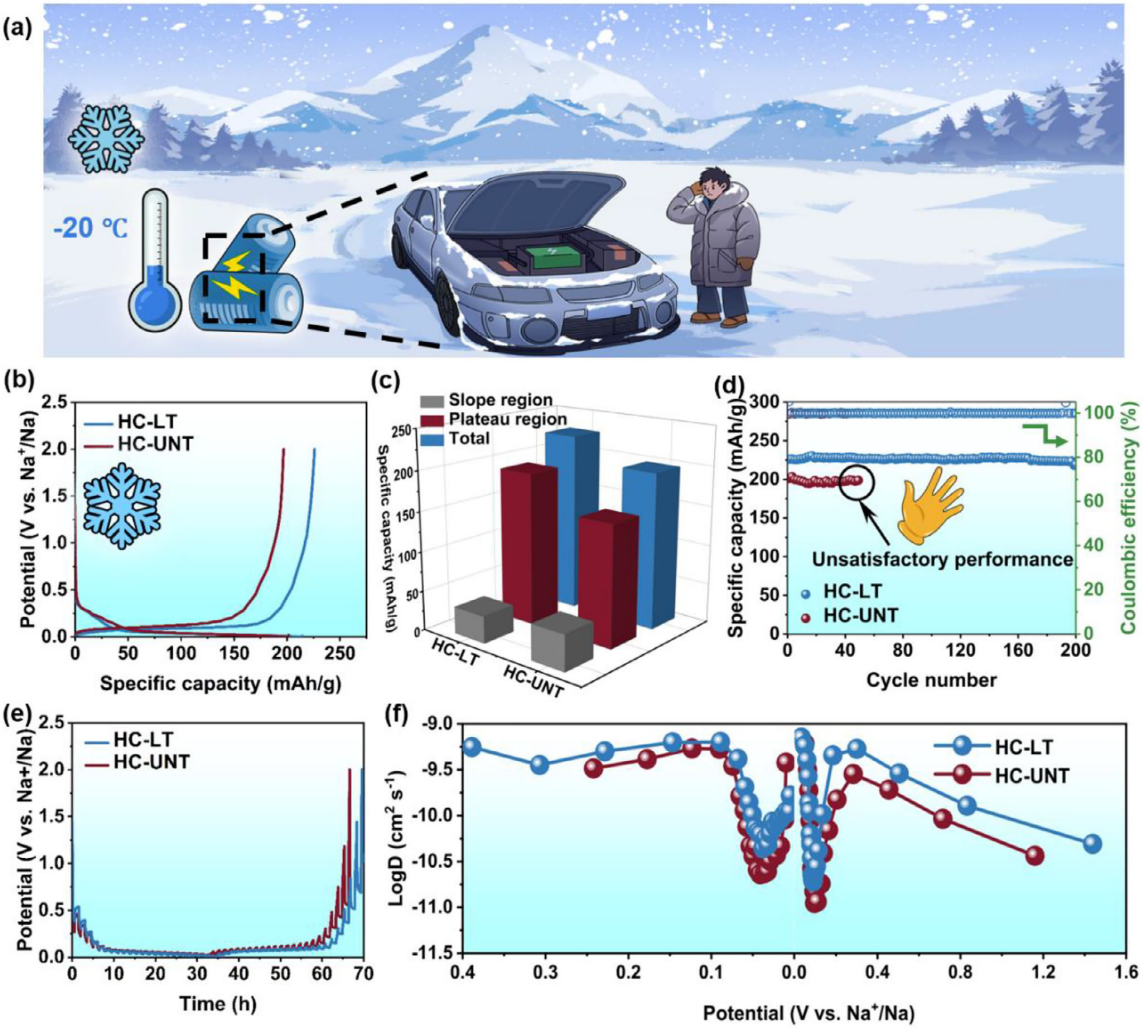
(a) Application of SIBs in low temperature environment. Low temperature performance (‐20°C), (b) GCD curves, (c) capacity proportion of different potential ranges, (d) cycle performance of the HC‐LT electrode and HC‐UNT electrode; (e) GITT potential profiles, and (f) the apparent ion diffusion coefficients.

SIBs exhibit great potential for low‐temperature application than lithium‐ion batteries, However, the diffusion kinetics within electrode materials at low temperatures remain impeded, presenting considerable challenges for practical deployment in cold climates (Figure 7a). Figure 7b illustrates the initial GCD profiles at ‐20°C. The HC‐LT electrode demonstrates a reversible capacity of 226.0 mAh/g, retaining 73.0% of its capacity at room temperature, which is notably higher than the 196.9 mAh/g observed for the HC‐UNT electrode. Furthermore, the HC‐LT electrode exhibits enhanced capacity within the plateau region and superior rate performance under low‐temperature conditions (Figure 7c; Figure S13). As shown in Figure 7d, the HC‐LT electrode exhibits stable cycling behavior over 200 cycles, with the charge capacity decreasing slightly from 226.0 to 220.4 mAh g^−1^, corresponding to an average fading rate of approximately 0.0124% per cycle, whereas the HC‐UNT electrode loses effective capacity after only 49 cycles. Low‐temperature kinetic analyses (Figure 7e,f) further reveal that the HC‐LT electrode possesses a higher sodium‐ion diffusion coefficient ($D_{Na^{+}}$). These excellent low‐temperature characteristics are attributed to the formation of graphitic‐like domains, which reduce charge transfer resistance, optimize interfacial reaction kinetics, and effectively mitigate kinetic degradation.

Additionally, as shown in Figure S14a,d, the charge‐transfer and ion‐diffusion kinetics of both electrodes were further analyzed using in situ EIS measurements under low‐temperature conditions. According to the time‐constant relation of a capacitive process: τ  =  *R* × *C* (where R is resistance and C is capacitance), a significant increase in R_ct_ at low temperatures leads to an increase in τ (since C changes more slowly than R). Consequently, the peak of the DRT shifts toward longer relaxation times (i.e., toward the right) at low temperatures. Moreover, HC‐LT shows a markedly smaller shift amplitude and a more gradual change in peak position (Figure S14b,e), which can be attributed to the optimized microcrystalline graphite structure. This phenomenon indicates that at low temperatures, the increase in the time constant corresponding to the interfacial charge transfer process is suppressed, leading to slower reaction kinetics. Meanwhile, HC‐LT maintains a relatively continuous and more reversible reaction kinetic timescale at low temperatures, directly demonstrating its low‐temperature performance advantage. Furthermore, in‐situ impedance fitting results at ‐20°C reveal (Figure S14c,d) that R_ct_ in hard carbon gradually decreases during discharge as Na^+^ inserts; subsequently, R_ct_ rebounds during charging, reflecting the dynamic evolution of interfacial charge transport and ion diffusion processes with reaction state under low‐temperature conditions. Moreover, HC‐LT with steric hindrance regulation consistently exhibits lower R_ct_ throughout the entire charge‐discharge cycle (Figure S14c,f), indicating superior interfacial charge transport kinetics at low temperatures. This advantage originates from the multiscale structural synergistic optimization induced by steric hindrance regulation, which effectively suppresses kinetic degradation and thereby underpins the excellent low‐temperature electrochemical performance of the material. Specifically, (i) by weakening the steric constraints imposed by highly crosslinked networks in the precursor, structural rearrangement is facilitated during carbonization, promoting the formation of microcrystalline graphite domains; and (ii) the accompanying structural reconstruction is also more favorable for the generation of closed‐pore structures. As a result, under low‐temperature conditions, HC‐LT benefits from a more optimized pore‐structure distribution and exhibits pronounced kinetic advantages: the microcrystalline graphite domains enhance electronic transport and reduce the interfacial charge‐transfer energy barrier, which is more effective in alleviating Na^+^ diffusion limitations at low temperature and mitigating the coupling constraints associated with interfacial reactions.

## 3 Conclusions

In summary, this work establishes and validates a steric‐hindrance‐based structural regulation strategy by elucidating the linkage between precursor molecular configuration and spatial steric effects. By weakening the steric constraints imposed by the 3D crosslinked lignin network and the complex hemicellulose structures, the restrictions during carbonization are reduced, enabling the predominantly linear cellulose‐derived carbon framework to undergo more sufficient rearrangement and stacking during pyrolysis. This, in turn, promotes the growth and curling of graphitic‐like microcrystalline domains. The structural evolution induced by steric hindrance alleviation not only suppresses the formation of excessively disordered carbon structures, but also favors the generation of topologically closed‐pore architectures. Benefiting from this regulation mechanism, the prepared hard carbon exhibits an increase in specific capacity from 261.2 mAh/g to 309.7 mAh/g, together with a high initial coulombic efficiency of 93.5%, achieving a synergistic improvement in sodium‐storage capacity and interfacial stability. Moreover, the material maintains a reversible capacity of 133.2 mAh/g at a high current density of 1000 mA/g, delivers excellent cycling stability over more than 7000 cycles at 400 mA/g, and demonstrates favorable low‐temperature performance (226.0 mAh/g at ‐20°C), collectively highlighting the positive role of precursor steric optimization in enhancing charge‐transfer kinetics and low‐temperature reaction reversibility. In addition, combined electrochemical measurements and in situ characterization confirm that the sodium‐storage mechanism of the hard carbon follows an “adsorption–insertion/pore‐filling” process.

## Conflicts of Interest

The authors declare no conflicts of interest.

## Supporting information




**Supporting File**: advs74653‐sup‐0001‐SuppMat.docx.

## Data Availability

The data that support the findings of this study are available from the corresponding author upon reasonable request.
