# Supplementary material for: Unlocking Plant‐Derived Potential: Regulating Microcrystalline Structure Design of High‐performance Hard Carbon Anodes via Cellulose Molecules
Source: Adv Sci (Weinh). 2026 Mar 9;13(27):e74653. doi: 10.1002/advs.74653 (PMC13170187; doi:10.1002/advs.74653)
Supplement: Supplementary file 1 — Supporting File: advs74653‐sup‐0001‐SuppMat.docx. [file ADVS-13-e74653-s001.docx]

**Supporting Information**

**Unlocking plant-derived potential: Regulating microcrystalline structure design of high-performance hard carbon anodes via cellulose molecules**

Xiping Zhang^a^, Wenhao Yang^a^, Dan You^a^, Ziyi Zhu^a*^, Xue Li^a*^

^1^National and Local Joint Engineering Research Center of Lithium-ion Batteries and Materials Preparation Technology, Key Laboratory of Advanced Battery Materials of Yunnan Province, Faculty of Metallurgical and Energy Engineering, Kunming University of Science and Technology, Kunming, China

**Corresponding author**

Ziyi Zhu: zyzhu23@kust.edu.cn

Xue Li: Lixue@kust.edu.cn

**1. Materials and methods**

1.1. Material synthesis

The waste green sandalwood powder was first sieved and subsequently subjected to heat treatment in a tube furnace at 1300 ℃ for 2 hours, hereafter referred to as HC-UNT. To optimize the microcrystalline structure, the precursor underwent the following pretreatment: First, green sandalwood powder was mixed with 2 g of NaClO_2_ (Aladdin, 80%) and 4 ml of CH_3_COOH (Aladdin, AR) in 300 ml of deionized water. The mixture was stirred at 80 ℃ for 6 hours, followed by filtration, washing, and drying. The dried product then underwent a hydrothermal reaction at 180 ℃ for 6 hours. After cooling, it was pulverized, filtered, washed, and dried. Finally, the material was subjected to the same pyrolysis process described above and designated HC-LT.

1.2. Material characterization

The types of functional groups of the two materials before and after treatment were analyzed by Fourier transform infrared spectroscopy (Thermo Fisher Scientific Nicolet iS20); the glass transition temperature of the samples was determined using simultaneous thermal analysis (Netzsch STA449F3, Germany).; the morphology of the samples was observed by scanning electron microscopy (Tescan-vega 3) and high resolution transmission electron microscopy (JEM2100); X-ray diffractometer (Mini Flex600) and confocal micro-Raman spectrometer (Horiba Labram Odyssey) were used to analyze the microstructure of the material; the Brunauer-Emmett-Teller method was used to process the isothermal adsorption-desorption curves and pore size distribution obtained by the American fully automatic specific surface area analyzers (Quantachrome Autosorb IQ3 and ASAP2020) under nitrogen (77 K) and CO_2_ (273.15 K) conditions, to obtain the corresponding specific surface area and pore structure information; the closed pore structure of the material was measured using a small angle X-ray scatterometer (Xenocs Xeuss 2.0); automatic true density/open and closed porosity analysis (Micromeritics AccuPyc II 1340) was used for true density testing.

1.3. Electrochemical measurements

Assemble the CR2016 on button battery cell in an argon-filled glove box. 94 wt.% active material, 1.5 wt.% Super-P (conductive agent), 1.5 wt.% CMC (binder) and 3 wt.% SBR (binder) were mixed, and deionized water was used as solvent to prepare uniform slurry. The slurry was coated on copper foil, dried in a vacuum oven at 60 ℃ for 12 h, and cut into electrodes with a diameter of 12 mm, corresponding to an active material loading of approximately ~3 mg. The electrode was assembled with sodium metal as the counter electrode, 1 M NaPF_6_-DEGDME as the electrolyte, and glass fiber as the separator. Cyclic voltammetry and electrochemical impedance spectroscopy were performed using an Autolab electrochemical workstation. The charging/discharging test is carried out on the LAND test system, and the voltage range is set to 0-2 V. The test parameters of the constant current intermittent titration method: the current pulse duration is 20 min, the relaxation time is 60 min, and the current density is 20 mA/g.

**2. Molecular Dynamics Simulations**

In order to reduce the dependence on molecular conformation in molecular modeling, the GAFF2 force field^[1]^ parameters are obtained using acpype code,^[2]^ and MMFF94 atomic charges are obtained by Open Babel software.^[3]^ Molecular dynamics simulations are performed using GROMACS 2021.7 software.^[4]^ Long-range electrostatic interactions are treated with the particle mesh Ewald (PME) method^[5]^ with 1.2 nm as the Coulomb cutoff, and the van der Waals (vdW) interactions are treated with the force-switching method,^[6]^ where the forces smoothly decayed to zero between 0.9 and 1.2 nm to reduce the cutoff noise. The LINCS algorithm^[7]^ is used to apply the bond constraint related to hydrogen, and a dispersion correction is used for both energy and pressure. All the simulation systems are energy-minimized by 10000 steps steepest descent with a time step of 1 fs, and then undergo 1 ns of NPT pre-equilibration run using leap-frog MD integrator^[8]^ before launching a long time (200 ns) unrestrained production run with a time step of 2 fs. During all the simulations, temperature is kept constant at 298.15K by using the velocity rescaling (V-rescale) thermostat^[9]^ (with *τ*_t_ = 2.0 ps), and pressure is kept at 1 bar by C-rescale isotropic barostat (with *τ*_p_ = 0.5 ps).^[10]^ The initial size of the simulated box is 8 nm×8 nm×8 nm and three-dimensional periodic boundary condition (PBC) is used. Results are visualized using VMD 1.9.3^[11]^ software.

**Figures:**


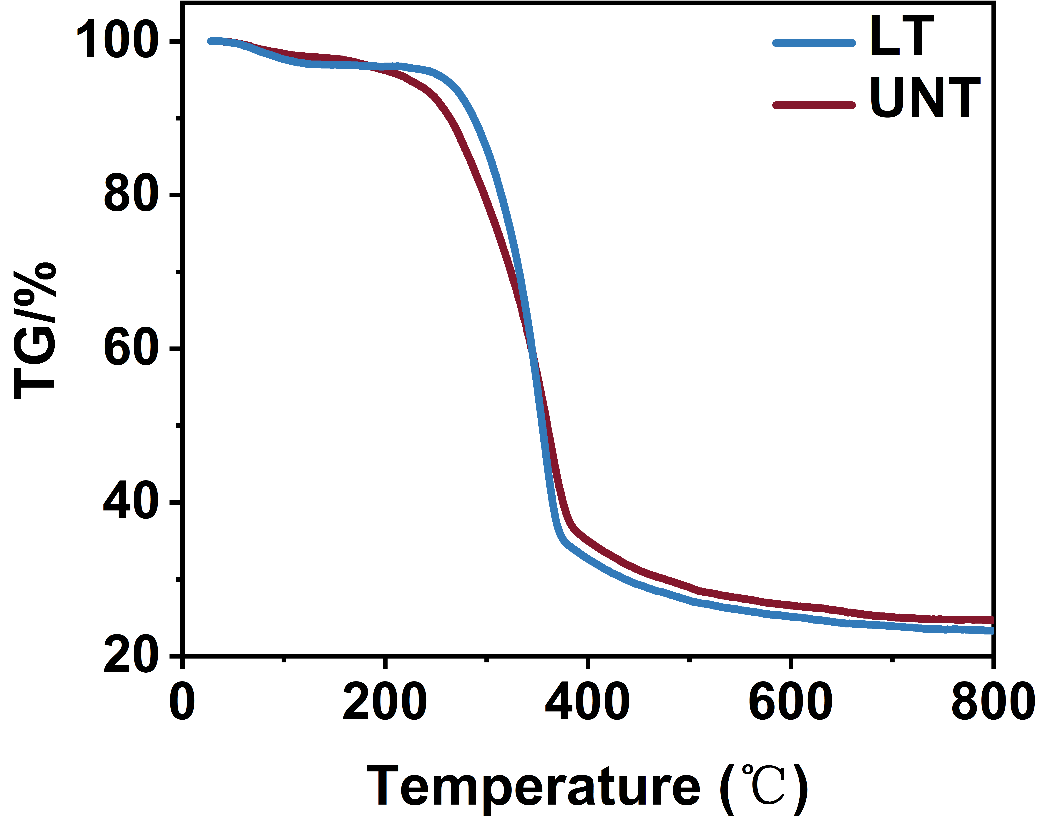


**Figure S1.** TG Curves of LT and UNT.


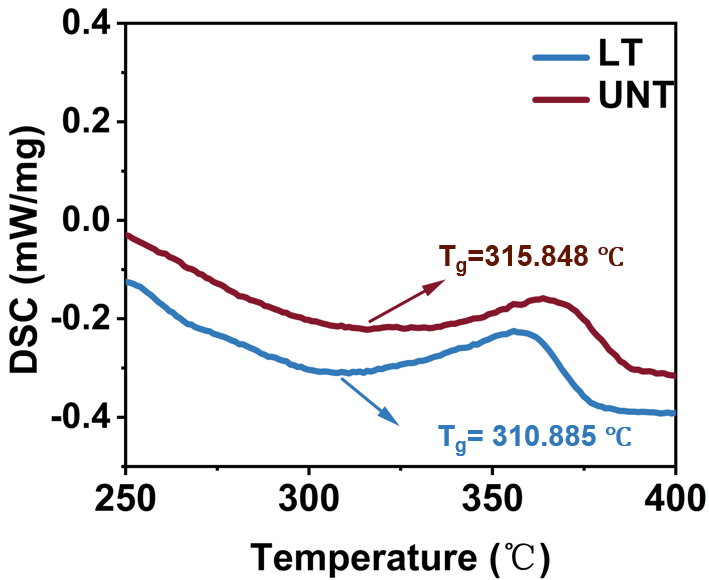


**Figure S2.** DSC Curves of LT and UNT.


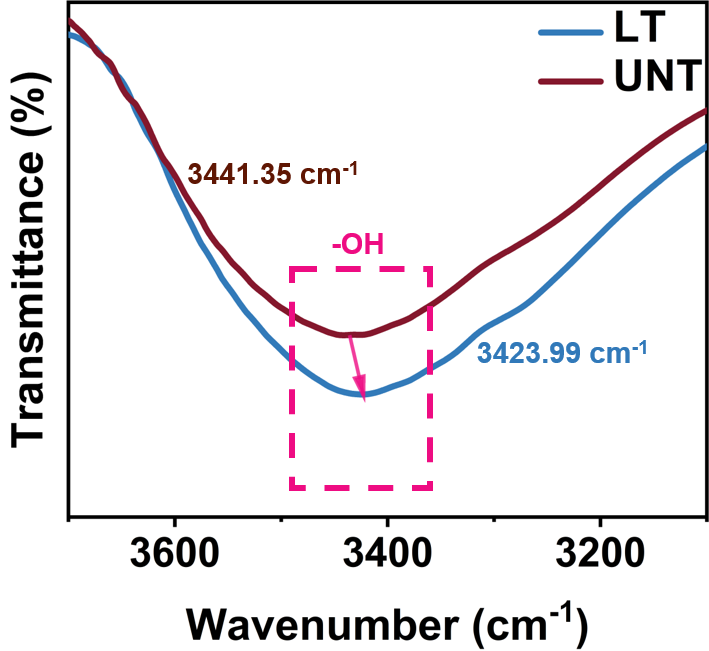


**Figure S3.** FTIR spectra.


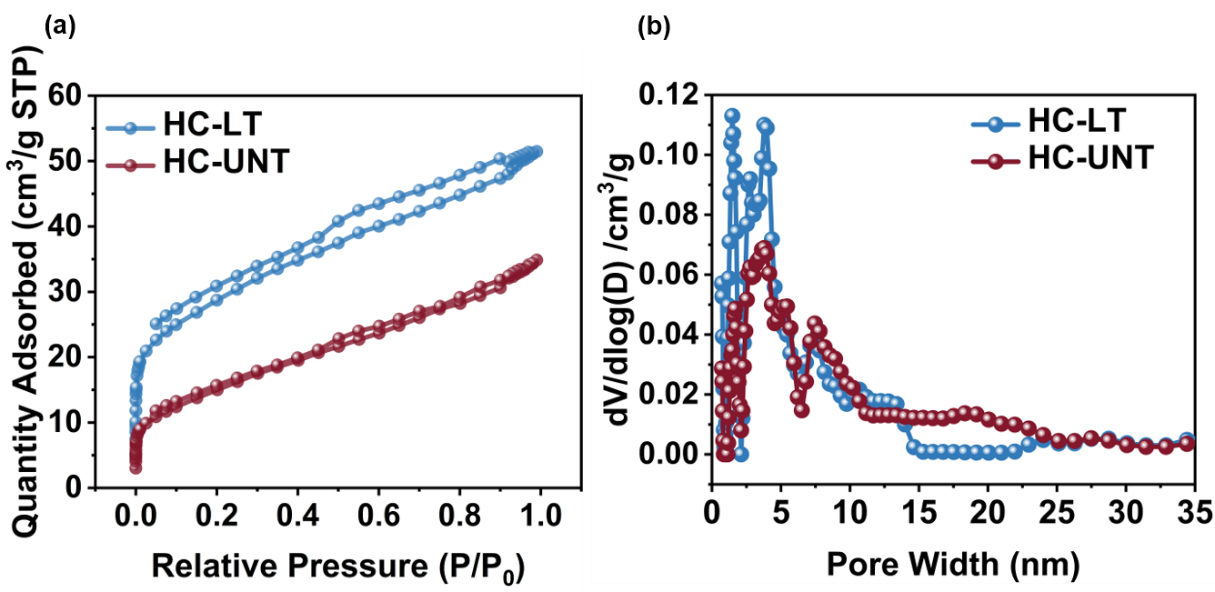


**Figure S4.** (a) N_2_ adsorption-desorption curves of HC-LT and HC-UNT. (b) Pore size distribution.


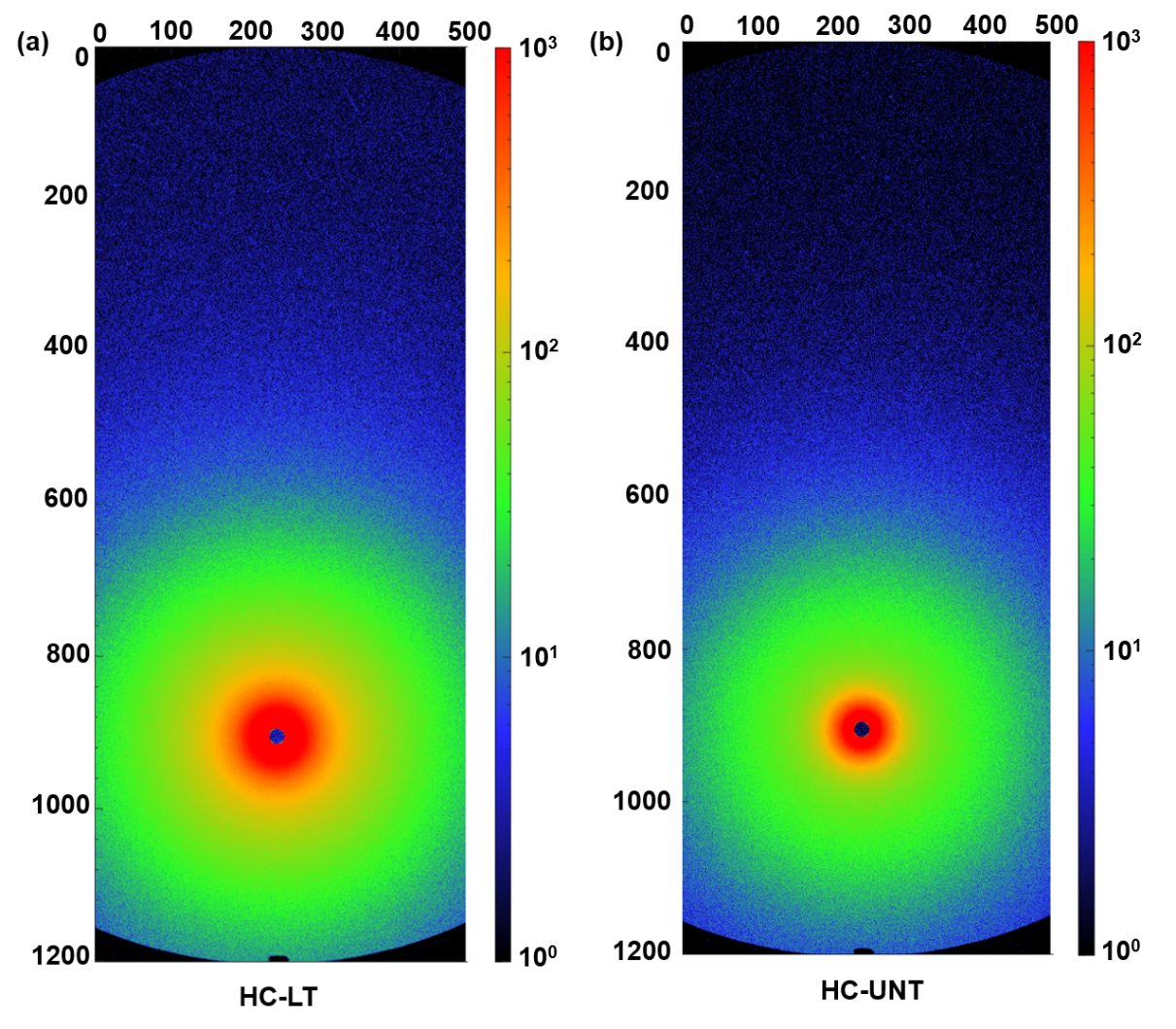


**Figure S5.** 2D SAXS of (a) HC-LT and (b) HC-UNT.


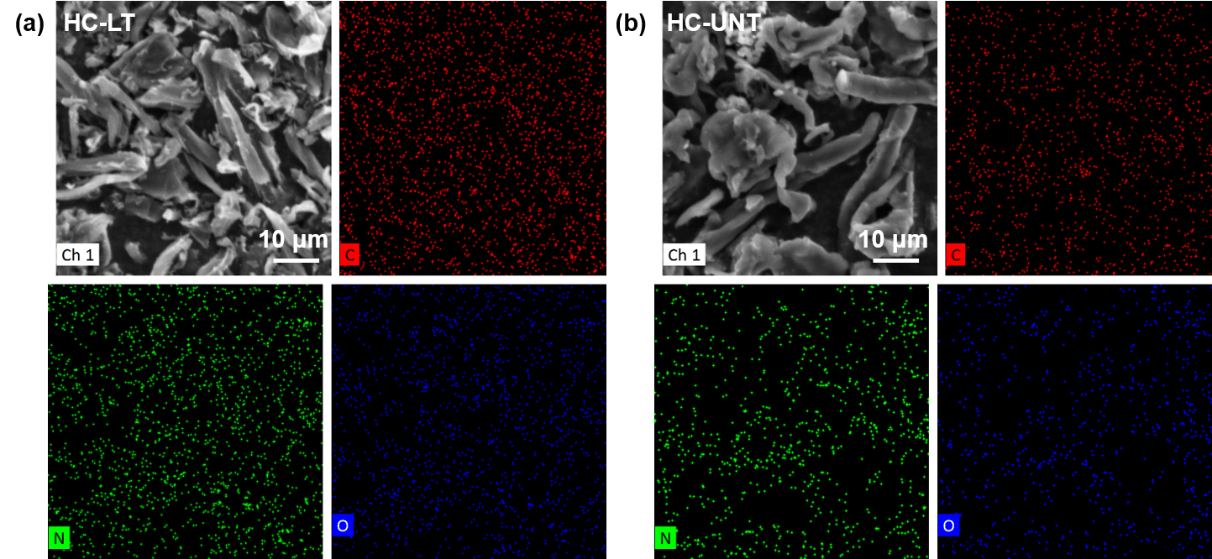


**Figure S6.** SEM and EDS images of (a)HC-LT and (b)HC-UNT.


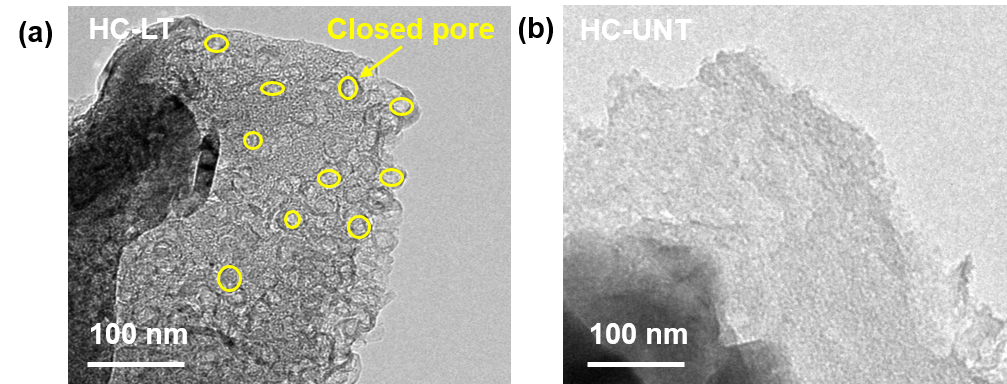


**Figure S7.** HRTEM images of (a) HC-LT and (b) HC-UNT.


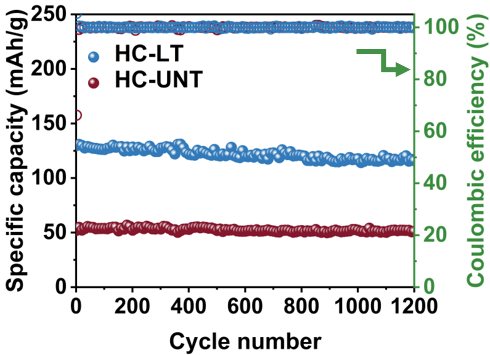


**Figure S8.** Cycle performance (1000 mA/g).


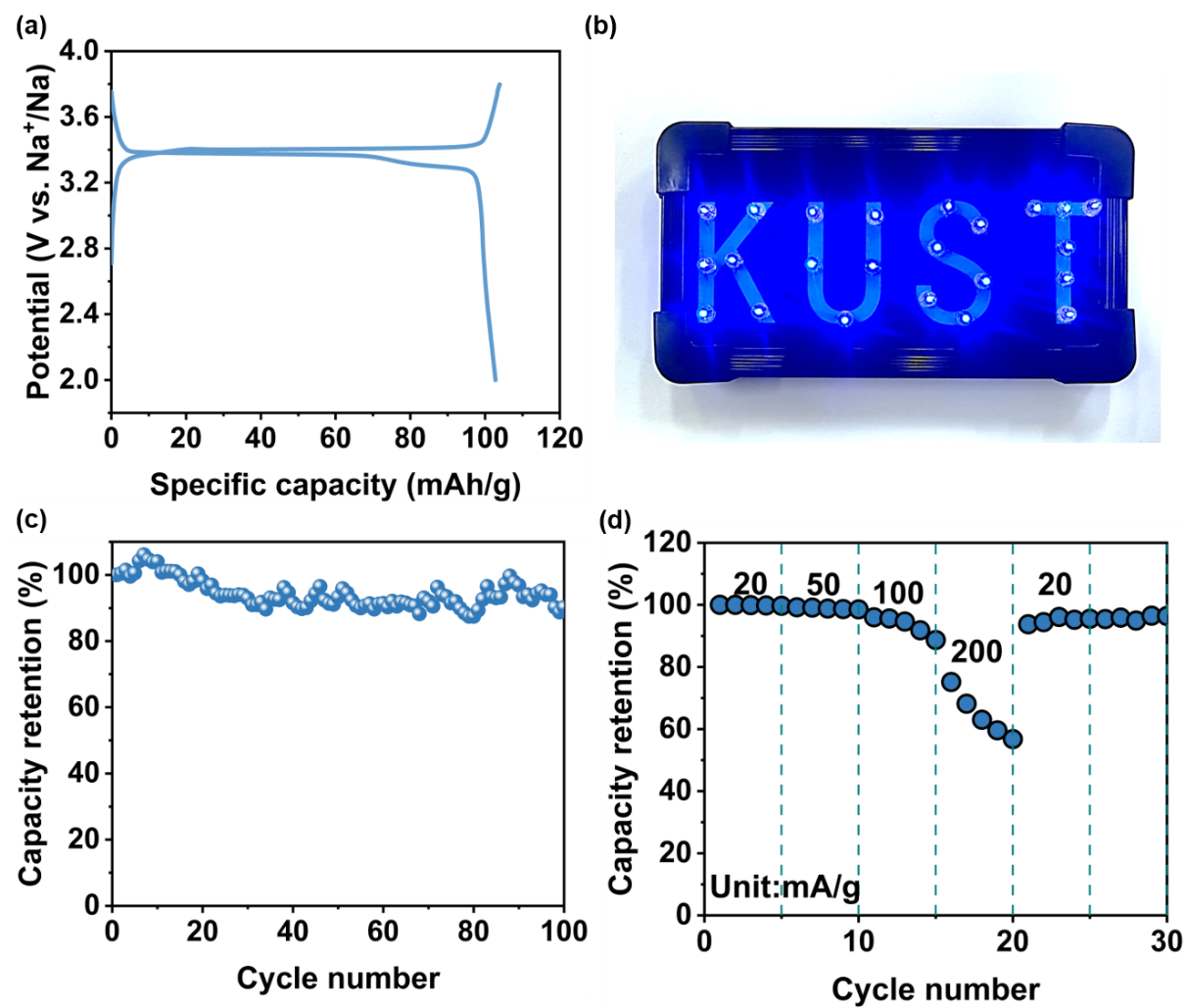


**Figure S9.** (a) Charge-discharge profiles of the full-cell. (b) Optical photograph illustrating LED lightened up by the full-cell. (c) Cycle performance and (d) Rate performance of the full-cell.

**
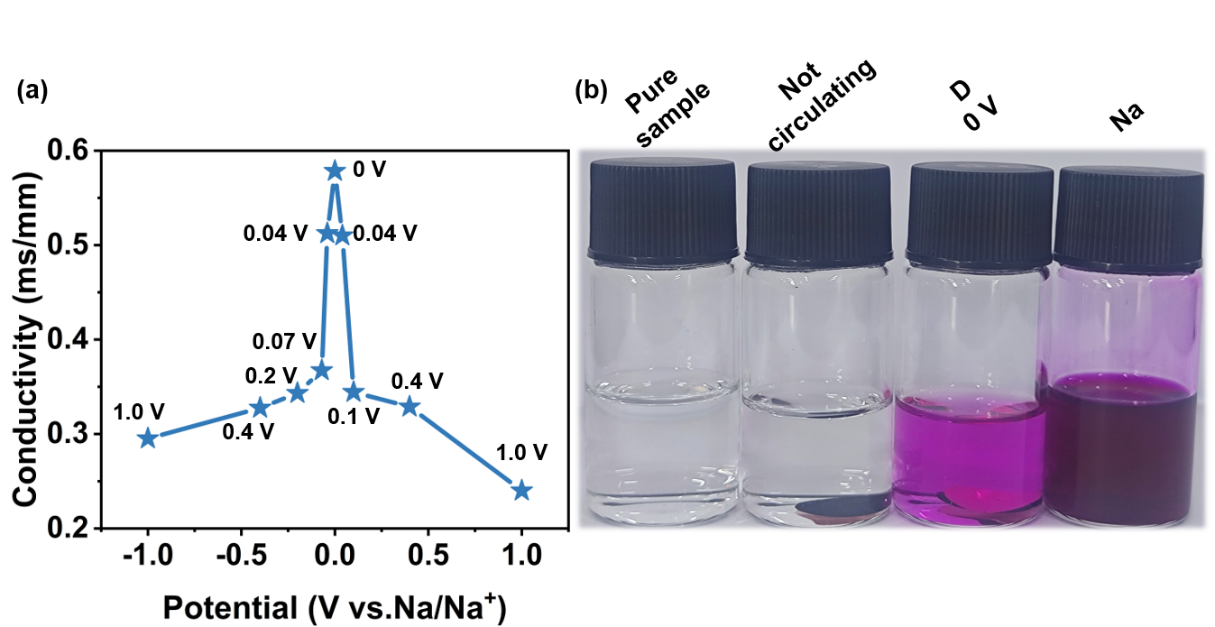
**

**Figure S10.** (a) Ionic conductivity of the ethanol solution at different potentials. (b) Color comparison of pure sample, fresh electrode, electrode discharged to 0 V and pure metal sodium in ethanol-phenolphthalein solution.

**
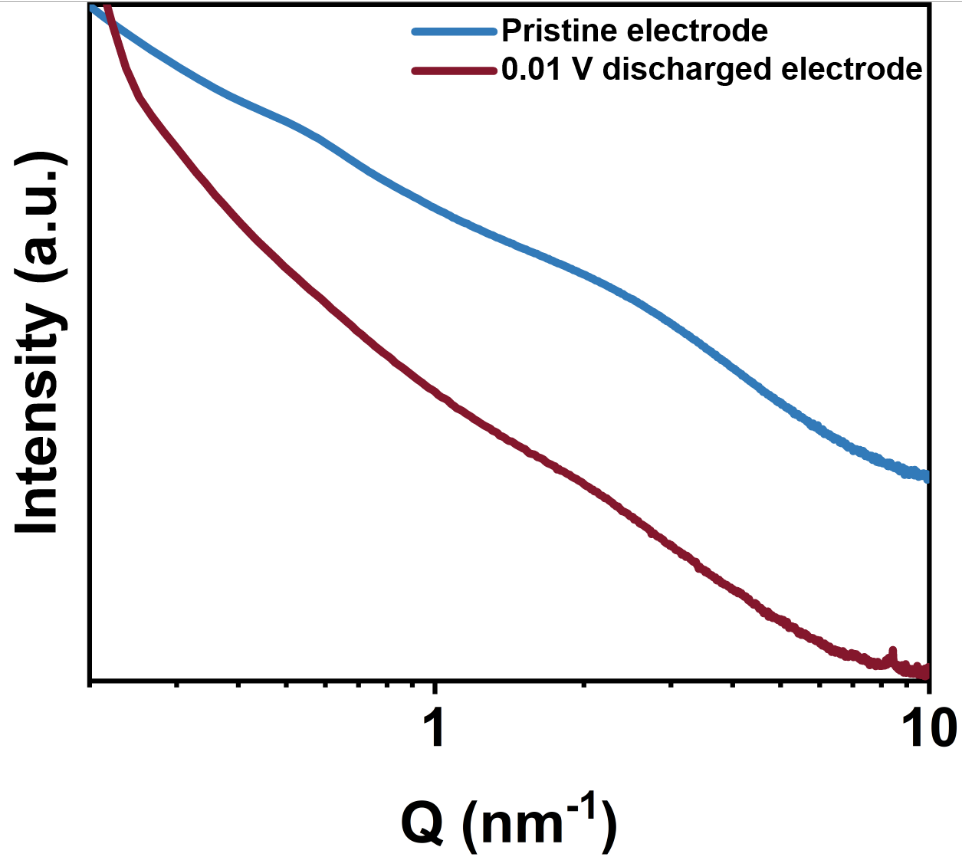
**

**Figure S11.** SAXS pattern of the HC-LT electrode after discharge to 0.01 V.


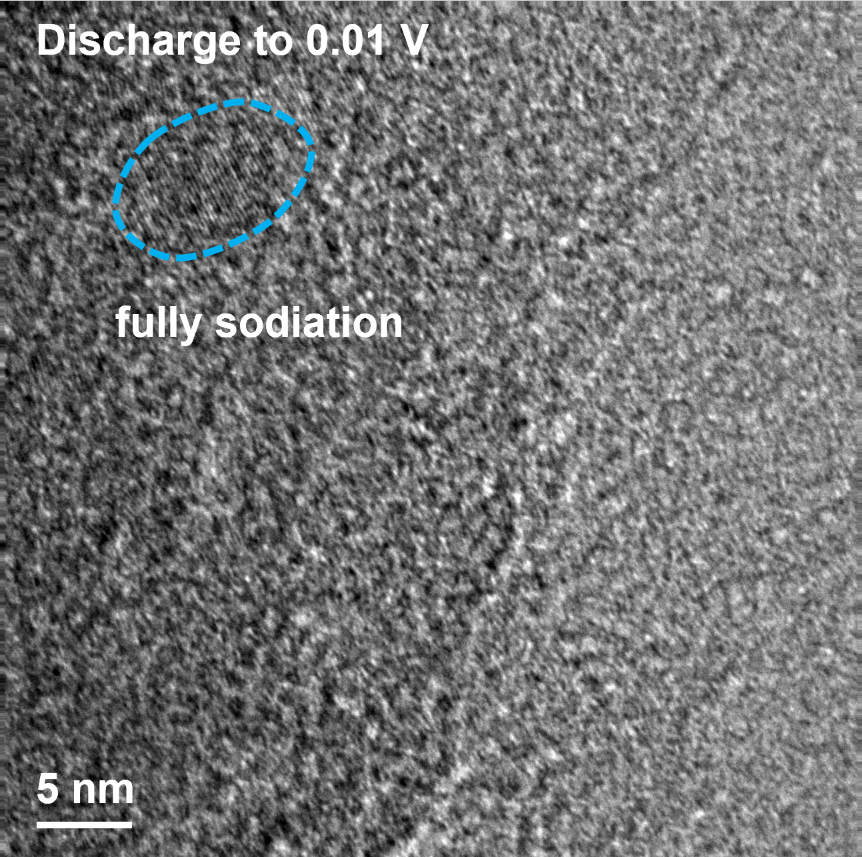


**Figure S12.** HRTEM image of fully sodiation material.


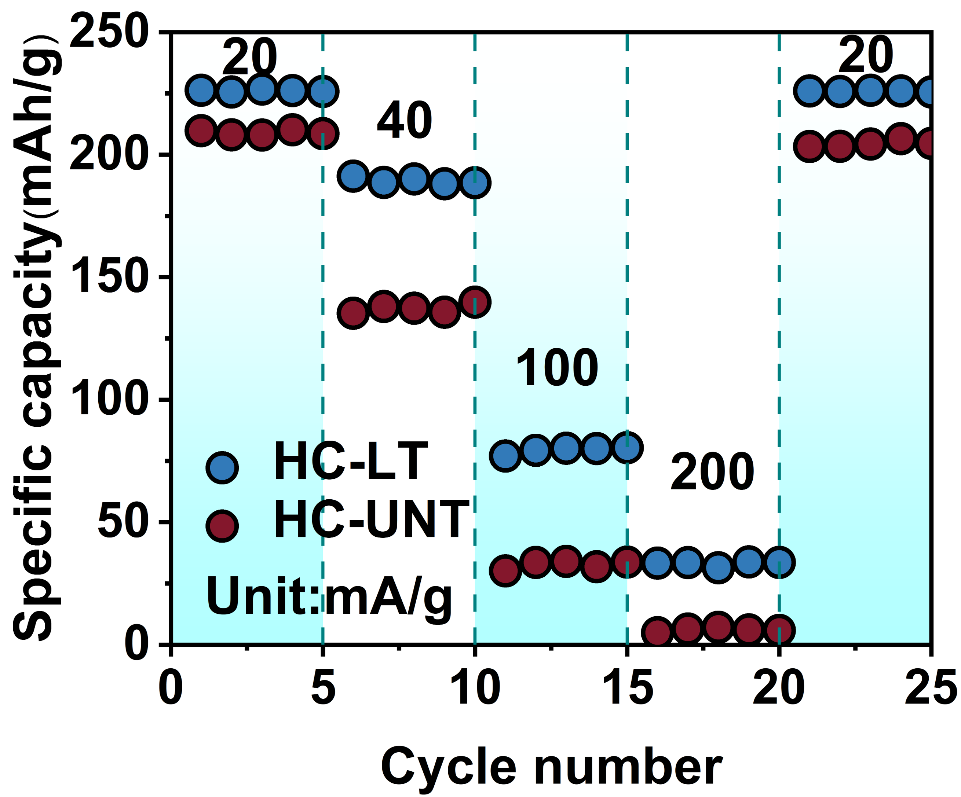


**Figure S13.** Rate performance (-20 ℃).

.


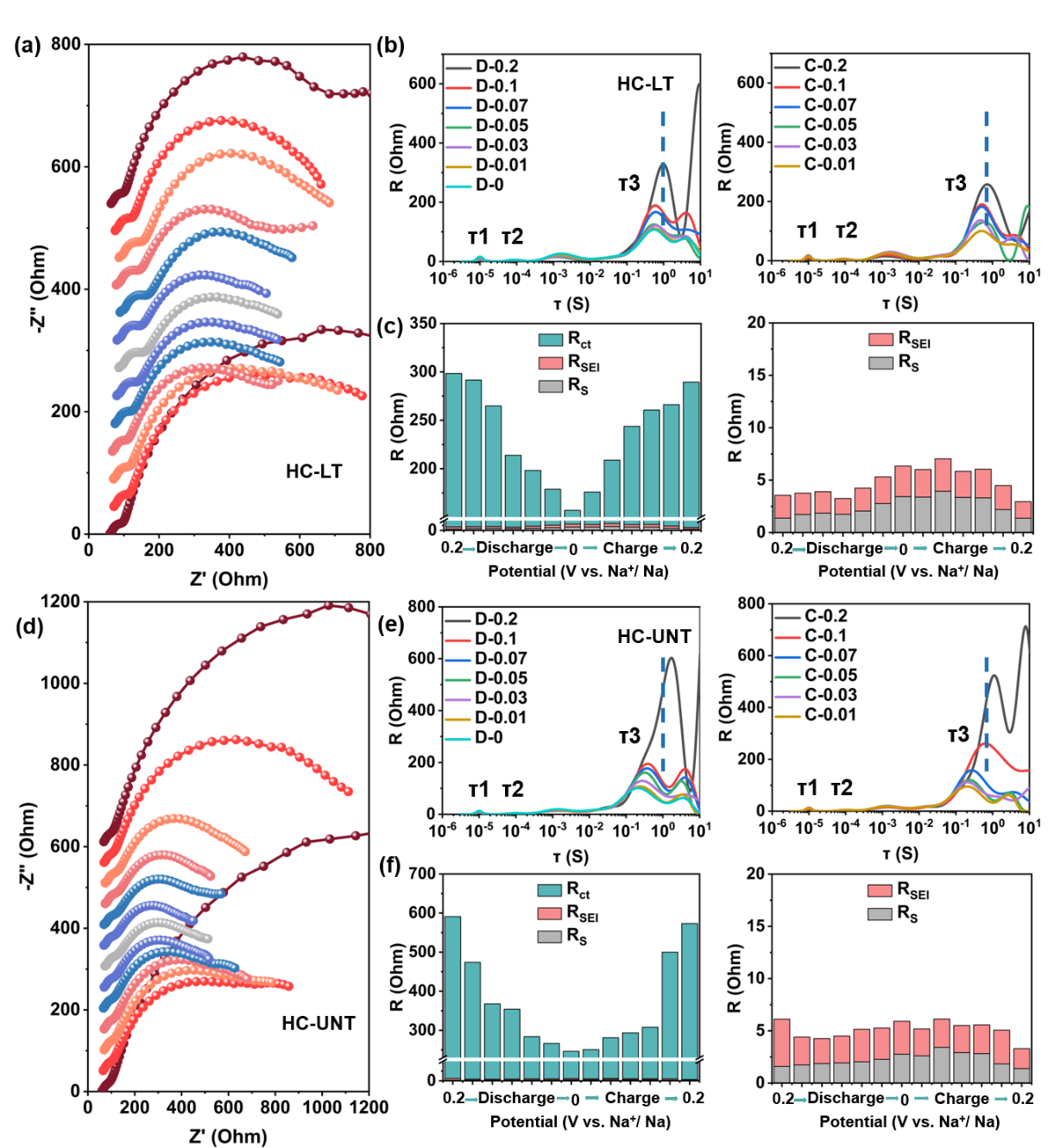


**Figure S14.** In situ impedance curves of (a) HC-LT electrode and (d) HC-UNT electrode. DRT spectra of (b) HC-LT electrode and (e) HC-UNT electrode. The corresponding resistance fitting results of (c) HC-LT electrode and (f) HC-UNT electrode.

**Tables:**

**Table S1.** Calculated free volumes of HC-UNT and HC-LT by GROMACS.

| Samples Units | HC-UNT | HC-LT |
| --- | --- | --- |
| Free volume (Å^3^) | 64456.191 | 61960.472 |

**Table S2.** L_a_ and L_c_ for HC-UNT and HC-LT.

| Samples Units | L_a_ | L_c_ |
| --- | --- | --- |
| HC-UNT | 2.2 | 1.2 |
| HC-LT | 2.6 | 1.0 |

**Table S3.** Comparison of electrochemical performance of hard carbon for SIBs

| **Precursor** | **Initial Coulombic efficiency** | **Reversible capacity** | **Rate performance** | **Cyclability** | **Low-temperature performance** | **Ref.** |
| --- | --- | --- | --- | --- | --- | --- |
| Lignin and cellulose | 87.1% | 343.3 mAh g^-1^ at 20 mA/g | 49.2 mAh/g at 1000 mA/g | below 90 mAh/g after 1000 cycles at 500 mA/g | — | [12] |
| Cellulose | 75.6% | 365.8 mAh/g at 20 mA/g | 62.9 mAh/g at 1000 mA/g | 98.0% after 100 cycles at 20 mA/g | — | [13] |
| Invasive alien plant | 52.2% | 285.0 mAh/g at 30 mA/g | 150 mAh/g at 1000 mA/g | 141.63 mAh/g after 1000 cycles at 200 mA/g | — | [14] |
| Sepals of Palmyra palm fruit calyx | 70.0% | 275.0 mAh g^-1^ at 30 mA/g | 131 mAh/g at 500 mA/g | 173 mAh/g after 100 cycles at 200 mA/g | — | [15] |
| Lotus | 50.4% | 330.6 mAh g^-1^ at 50 mA/g | 78.3 mAh/g at 1000 mA/g | 161.5 mAh/g after 500 cycles at 200 mA/g | — | [16] |
| Chickpea | — | 330 mAh/g at 20 mA/g | 90 mAh/g at 1000 mA/g | 89.5% after 500 cycles at 1000 mA/g | — | [17] |
| Glucose | 72.1% | 481.5 mAh/g at 20 mA/g | 58.4 mAh/g at 1000 mA/g | 91.5% after 100 cycles at 500 mA/g | — | [18] |
| Polypropylene | 88.7 % | 335.5 mAh/g at 30 mA/g | 69.8 mAh/g at 600 mA/g | 79.6 mAh/g after 500 cycles at 300 mA/g | — | [19] |
| Anthracite | 86.9% | 332.0 mAh/g at 0.1 C | 193 mAh/g at 2 C | 280 mAh/g after 500 cycles at 0.3 C | 202 mAh/g at -20 ℃ | [20] |
| Longan Peel | 80% | 309.0 mAh/g at 25 mA/g | below 100 mAh/g at 1000 mA/g | — | 250 mAh/g at -20 ℃ | [21] |
| Coal | 86.4% | 365.8 mAh/g at 20 mA/g | 38.4 mAh/g at 600 mA/g | 97.7% after 50 cycles at 20 mA/g | 185.4 mAh/g at -20 ℃ | [22] |
| **Wood** | **93.5%** | **309.7 mAh/g at 20 mA/g** | **133.2 mAh/g at 1000 mA/g** | **80.5% after 7000 cycles (199.4 mAh/g) at 400 mA/g** | **226.0 mAh/g at -20 ℃** | **★This Work** |

**References**

[1] J. Wang, R. M. Wolf, J. W. Caldwell, P. A. Kollman, D. A. Case, *J. Comput. Chem.* **2004**, *25*, 1157.

[2] A. W. Sousa Da Silva, W. F. Vranken, *BMC Res. Notes* **2012**, *5*.

[3] N. M. O’Boyle, M. Banck, C. A. James, C. Morley, T. Vandermeersch, G. R. Hutchison, *J. Cheminformatics* **2011**, *3*.

[4] M. J. Abraham, T. Murtola, R. Schulz, S. Páll, J. C. Smith, B. Hess, E. Lindahl, *SoftwareX* **2015**, *1–2*, 19.

[5] U. Essmann, L. Perera, M. L. Berkowitz, T. Darden, H. Lee, L. G. Pedersen, *J. Chem. Phys.* **1995**, *103*, 8577.

[6] P. J. Steinbach, B. R. Brooks, *J. Comput. Chem.* **1994**, *15*, 667.

[7] B. Hess, H. Bekker, H. J. C. Berendsen, J. G. E. M. Fraaije, *J. Comput. Chem.* **1997**, *18*, 1463.

[8] R. W. Hockney, S. P. Goel, J. W. Eastwood, *J. Comput. Phys.* **1974**, *14*, 148.

[9] G. Bussi, D. Donadio, M. Parrinello, *J. Chem. Phys.* **2007**, *126*.

[10] M. Bernetti, G. Bussi, *J. Chem. Phys.* **2020**, *153*.

[11] W. Humphrey, A. Dalke, K. Schulten, *J. Mol. Graph.* **1996**, *14*, 33.

[12] X.-S. Wu, X.-L. Dong, B.-Y. Wang, J.-L. Xia, W.-C. Li, *Renew. Energy* **2022**, *189*, 630.

[13] B. Zhao, X. Li, L. Shang, C. Qiu, R. Yuan, H. Liu, T. Liu, A. Li, X. Chen, H. Song, *J. Mater. Chem. A* **2024**, *12*, 5834.

[14] H. Wei, H. Cheng, N. Yao, G. Li, Z. Du, R. Luo, Z. Zheng, *Chemosphere* **2023**, *343*, 140220.

[15] D. Damodar, S. Ghosh, M. Usha Rani, S. K. Martha, A. S. Deshpande, *J. Power Sources* **2019**, *438*, 227008.

[16] F. Wu, M. Zhang, Y. Bai, X. Wang, R. Dong, C. Wu, *ACS Appl. Mater. Interfaces* **2019**, *11*, 12554.

[17] U. Ghani, N. Iqbal, A. A. Aboalhassan, B. Liu, T. Aftab, I. Zada, F. Ullah, J. Gu, Y. Li, S. Zhu, Q. Liu, *J. Colloid Interface Sci.* **2022**, *611*, 578.

[18] C. Qiu, A. Li, D. Qiu, Y. Wu, Z. Jiang, J. Zhang, J. Xiao, R. Yuan, Z. Jiang, X. Liu, X. Chen, H. Song, *ACS Nano* **2024**, *18*, 11941.

[19] S. Zhang, N. Sun, X. Li, R. A. Soomro, B. Xu, *Energy Storage Mater.* **2024**, *66*, 103183.

[20] R. Li, C. Su, W. Yang, R. Xu, K. Chen, J. Chen, W. Su, B. Yuan, J. Long, A. Hu, *Chem. Eng. J.* **2025**, *506*, 159968.

[21] X. Lin, X. Du, P. S. Tsui, J.-Q. Huang, H. Tan, B. Zhang, *Electrochimica Acta* **2019**, *316*, 60.

[22] R. Yang, X. Qiu, H. Xiong, Z. Cui, Y. Xue, R. Zhou, J. Jiang, X. Kong, L. Dong, Q. Zhuang, Z. Ju, Y. Chen, *Energy Storage Mater.* **2025**, *80*, 104415.
